# Robustness Property of Robust-BD Wald-Type Test for Varying-Dimensional General Linear Models

**DOI:** 10.3390/e20030168

**Published:** 2018-03-05

**Authors:** Xiao Guo, Chunming Zhang

**Affiliations:** 1 Department of Statistics and Finance, School of Management, University of Science and Technology of China, Hefei 230026, China; 2 Department of Statistics, University of Wisconsin-Madison, Madison, WI 53706, USA

**Keywords:** Bregman divergence, general linear model, hypothesis testing, influence function, robust, Wald-type test

## Abstract

An important issue for robust inference is to examine the stability of the asymptotic level and power of the test statistic in the presence of contaminated data. Most existing results are derived in finite-dimensional settings with some particular choices of loss functions. This paper re-examines this issue by allowing for a diverging number of parameters combined with a broader array of robust error measures, called “*robust-BD*”, for the class of “general linear models”. Under regularity conditions, we derive the influence function of the *robust-BD* parameter estimator and demonstrate that the *robust-BD* Wald-type test enjoys the robustness of validity and efficiency asymptotically. Specifically, the asymptotic level of the test is stable under a small amount of contamination of the null hypothesis, whereas the asymptotic power is large enough under a contaminated distribution in a neighborhood of the contiguous alternatives, thus lending supports to the utility of the proposed *robust-BD* Wald-type test.

## 1. Introduction

The class of varying-dimensional “general linear models” [1], including the conventional generalized linear model (GLM in [2]), is flexible and powerful for modeling a large variety of data and plays an important role in many statistical applications. In the literature, it has been extensively studied that the conventional maximum likelihood estimator for the GLM is nonrobust; for example, see [3,4]. To enhance the resistance to outliers in applications, many efforts have been made to obtain robust estimators. For example, Noh et al. [5] and Künsch et al. [6] developed robust estimator for the GLM, and Stefanski et al. [7], Bianco et al. [8] and Croux et al. [9] studied robust estimation for the logistic regression model with the deviance loss as the error measure.

Besides robust estimation for the GLM, robust inference is another important issue, which, however, receives relatively less attention. Basically, the study of robust testing includes two aspects: (i) establishing the stability of the asymptotic level under small departures from the null hypothesis (i.e., robustness of “validity”); and (ii) demonstrating that the asymptotic power is sufficiently large under small departures from specified alternatives (i.e., robustness of “efficiency”). In the literature, robust inference has been conducted for different models. For example, Heritier et al. [10] studied the robustness properties of the Wald, score and likelihood ratio tests based on M estimators for general parametric models. Cantoni et al. [11] developed a test statistic based on the robust deviance, and conducted robust inference for the GLM using quasi-likelihood as the loss function. A robust Wald-type test for the logistic regression model is studied in [12]. Ronchetti et al. [13] concerned the robustness property for the generalized method of moments estimators. Basu et al. [14] proposed robust tests based on the density power divergence (DPD) measure for the equality of two normal means. Robust tests for parameter change have been studied using the density-based divergence method in [15,16]. However, the aforementioned methods based on the GLM mostly focus on situations where the number of parameters is fixed and the loss function is specific.

Zhang et al. [1] developed robust estimation and testing for the “general linear model” based on a broader array of error measures, namely Bregman divergence, allowing for a diverging number of parameters. The Bregman divergence includes a wide class of error measures as special cases, e.g., the (negative) quasi-likelihood in regression, the deviance loss and exponential loss in machine learning practice, among many other commonly used loss functions. Zhang et al. [1] studied the consistency and asymptotic normality of their proposed *robust-BD* parameter estimator and demonstrated the asymptotic distribution of the Wald-type test constructed from *robust-BD* estimators. Naturally, it remains an important issue to examine the robustness property of the *robust-BD* Wald-type test [1] in the varying-dimensional case, i.e., whether the test still has stable asymptotic level and power, in the presence of contaminated data.

This paper aims to demonstrate the robustness property of the *robust-BD* Wald-type test in [1]. Nevertheless, it is a nontrivial task to address this issue. Although the local stability for the Wald-type tests have been established for the M estimators [10], generalized method of moment estimators [13], minimum density power divergence estimator [17] and general M estimators under random censoring [18], their results for finite-dimensional settings are not directly applicable to our situations with a diverging number of parameters. Under certain regularity conditions, we provide rigorous theoretical derivation for robust testing based on the Wald-type test statistic. The essential results are approximations of the asymptotic level and power under contaminated distributions of the data in a small neighborhood of the null and alternative hypotheses, respectively.

- Specifically, we show in Theorem 1 that, if the influence function of the estimator is bounded, then the asymptotic level of the test is also bounded under a small amount of contamination.
- We also demonstrate in Theorem 2 that, if the contamination belongs to a neighborhood of the contiguous alternatives, then the asymptotic power is also stable.

Hence, we contribute to establish the robustness of validity and efficiency for the *robust-BD* Wald-type test for the “general linear model” with a diverging number of parameters.

The rest of the paper is organized as follows. Section 2 reviews the Bregman divergence (BD), *robust-BD* estimation and the Wald-type test statistic proposed in [1]. Section 3 derives the influence function of the *robust-BD* estimator and studies the robustness properties of the asymptotic level and power of the Wald-type test under a small amount of contamination. Section 4 conducts the simulation studies. The technical conditions and proofs are given in Appendix A. A list of notations and symbols is provided in Appendix B.

We will introduce some necessary notations. In the following, *C* and *c* are generic finite constants which may vary from place to place, but do not depend on the sample size *n*. Denote by $\;E_{K}\left(\cdot \right)$ the expectation with respect to the underlying distribution *K*. For a positive integer *q*, let $0_{q}={\left(0,\ldots ,0\right)}^{T}\in R^{q}$ be a q×1 zero vector and $I_{q}$ be the q×q identity matrix. For a vector $v={\left(v_{1},\ldots ,v_{q}\right)}^{T}\in R^{q}$, the $L_{1}$ norm is ${∥v∥}_{1}=\sum_{i=1}^{q}\left|v_{i}\right|$, $L_{2}$ norm is ${∥v∥}_{2}={\left(\sum_{i=1}^{q}v_{i}^{2}\right)}^{1/2}$ and the $L_{\infty }$ norm is ${∥v∥}_{\infty }=\max_{i=1,\ldots ,q}\left|v_{i}\right|$. For a q×q matrix *A*, the $L_{2}$ and Frobenius norms of *A* are ${∥A∥}_{2}={\left\{\lambda_{\max}\left(A^{T}A\right)\right\}}^{1/2}$ and ${∥A∥}_{F}=\sqrt{\mathrm{tr}(AA^{T})}$, respectively, where $\lambda_{\max}\left(\cdot \right)$ denotes the largest eigenvalue of a matrix and tr(·) denotes the trace of a matrix.

## 2. Review of Robust-BD Estimation and Inference for “General Linear Models”

This section briefly reviews the *robust-BD* estimation and inference methods for the “general linear model” developed in [1]. Let $\left\{\left(X_{n1},Y_{1}\right),\ldots ,\left(X_{nn},Y_{n}\right)\right\}$ be i.i.d. observations from some underlying distribution $\left(X_{n},Y\right)$ with $X_{n}={\left(X_{1},\ldots ,X_{p_{n}}\right)}^{T}\in R^{p_{n}}$ the explanatory variables and *Y* the response variable. The dimension $p_{n}$ is allowed to diverge with the sample size *n*. The “general linear model” is given by

(1) $$\begin{array}{c} m\left(x_{n}\right)\equiv \;E\left(Y|X_{n}=x_{n}\right)=F^{-1}\left({\tilde{x}}_{n}^{T}{\tilde{\beta }}_{n,0}\right), \end{array}$$

and

(2) $$\begin{array}{c} \mathrm{var}\left(Y|X_{n}=x_{n}\right)=V\left(m\left(x_{n}\right)\right), \end{array}$$

where *F* is a known link function, ${\tilde{\beta }}_{n,0}\in R^{p_{n}+1}$ is the vector of unknown true regression parameters, ${\tilde{x}}_{n}={\left(1,x_{n}^{T}\right)}^{T}$ and V(·) is a known function. Note that the conventional generalized linear model (GLM) satisfying Equations (1) and (2) assumes that $Y|X_{n}=x_{n}$ follows a particular distribution in the exponential family. However, our “general linear model” does not require explicit form of distributions of the response. Hence, the “general linear model” includes the GLM as a special case. For notational simplicity, denote $Z_{n}={\left(X_{n}^{T},Y\right)}^{T}$ and ${\tilde{Z}}_{n}={\left({\tilde{X}}_{n}^{T},Y\right)}^{T}$.

Bregman divergence (BD) is a class of error measures, which is introduced in [19] and covers a wide range of loss functions. Specifically, Bregman divergence is defined as a bivariate function,

$$\begin{array}{c} Q_{q}\left(\nu ,\mu \right)=-q\left(\nu \right)+q\left(\mu \right)+\left(\nu -\mu \right)q^{\prime }\left(\mu \right), \end{array}$$

where q(·) is the concave generating *q*-function. For example, $q\left(\mu \right)=a\mu -\mu^{2}$ for a constant *a* corresponds to the quadratic loss $Q_{a}\left(Y,\mu \right)={\left(Y-\mu \right)}^{2}$. For a binary response variable *Y*, q(μ)=min{μ,1−μ} gives the misclassification loss $Q_{q}\left(Y,\mu \right)=I\left\{Y\neq I\left(\mu >0.5\right)\right\}$; q(μ)=−2{μlog(μ)+(1−μ)log(1−μ)} gives Bernoulli deviance loss $Q_{q}\left(Y,\mu \right)=-2\left\{Y\log\left(\mu \right)+\left(1-Y\right)\log\left(1-\mu \right)\right\}$; q(μ)=2min{μ,1−μ} gives the hinge loss $Q_{q}\left(Y,\mu \right)=\max\left\{1-\left(2Y-1\right)\mathrm{sign}\left(\mu -0.5\right),0\right\}$ for the support vector machine; $q\left(\mu \right)=2{\left\{\mu \left(1-\mu \right)\right\}}^{1/2}$ yields the exponential loss $Q_{q}\left(Y,\mu \right)=\exp\left[-\left(Y-0.5\right)\log\left\{\mu /\left(1-\mu \right)\right\}\right]$ used in AdaBoost [20]. Furthermore, Zhang et al. [21] showed that if

(3) $$\begin{array}{c} q\left(\mu \right)=\int_{a}^{\mu }\frac{s-\mu }{V(s)}ds, \end{array}$$

where *a* is a finite constant such that the integral is well-defined, then $Q_{q}\left(y,\mu \right)$ is the “*classical (negative) quasi-likelihood*” function $-Q_{\mathrm{QL}}\left(y,\mu \right)$ with $\partial Q_{\mathrm{QL}}\left(y,\mu \right)/\partial \mu =\left(y-\mu \right)/V\left(\mu \right)$.

To obtain a robust estimator based on BD, Zhang et al. [1] developed the *robust-BD* loss function

(4) $$\begin{array}{c} \rho_{q}\left(y,\mu \right)=\int_{y}^{\mu }\psi \left(r\left(y,s\right)\right)\left\{q^{\prime \prime }\left(s\right)\sqrt{V(s)}\right\}ds-G\left(\mu \right), \end{array}$$

where ψ(·) is a bounded odd function, such as the Huber ψ-function [22], $r\left(y,s\right)=\left(y-s\right)/\sqrt{V(s)}$ denotes the Pearson residual and G(μ) is the bias-correction term satisfying

$$\begin{array}{c} G^{\prime }\left(\mu \right)=G_{1}^{\prime }\left(\mu \right)\left\{q^{\prime \prime }\left(\mu \right)\sqrt{V(\mu )}\right\}, \end{array}$$

with

$$\begin{array}{c} G_{1}^{\prime }\left(m\left(x_{n}\right)\right)=\;E\left\{\psi \left(r\left(Y,m\left(x_{n}\right)\right)\right)|X_{n}=x_{n}\right\}. \end{array}$$

Based on *robust-BD*, the estimator of ${\tilde{\beta }}_{n,0}$ proposed in [1] is defined as

(5) $$\begin{array}{c} \hat{\tilde{\beta }}=\arg\min_{\tilde{\beta }}\left\{\frac{1}{n}\sum_{i=1}^{n}\rho_{q}\left(Y_{i},F^{-1}\left({\tilde{X}}_{ni}^{T}\tilde{\beta }\right)\right)w\left(X_{ni}\right)\right\}, \end{array}$$

where w(·)≥0 is a known bounded weight function which downweights the high leverage points.

In [11], the “*robust quasi-likelihood estimator*” of ${\tilde{\beta }}_{n,0}$ is formulated according to the “*robust quasi-likelihood function*” defined as

$$\begin{array}{cc} & Q_{\mathrm{RQL}}\left(x_{n},y,\mu \right)\\ = & \left\{\int_{\mu_{0}}^{\mu }\psi \left(r\left(y,s\right)\right)/\sqrt{V(s)}ds\right\}w\left(x_{n}\right)-\frac{1}{n}\sum_{j=1}^{n}\int_{\mu_{0}}^{\mu_{j}}\left[\;E\left\{\psi \left(r\left(Y_{j},s\right)\right)|X_{nj}\right\}/\sqrt{V(s)}ds\right]w\left(X_{nj}\right), \end{array}$$

where $\mu =F^{-1}\left({\tilde{x}}_{n}^{T}\tilde{\beta }\right)$ and $\mu_{j}=\mu_{j}\left(\tilde{\beta }\right)=F^{-1}\left({\tilde{X}}_{nj}^{T}\tilde{\beta }\right)$, j=1,…,n. To describe the intuition of the “*robust-BD*”, we use the following diagram from [1], which illustrates the relation among the “*robust-BD*”, “*classical-BD*”, “*robust quasi-likelihood*” and “*classical (negative) quasi-likelihood*”.


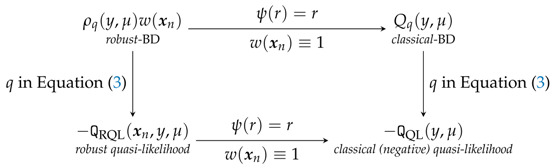


For the *robust-BD*, assume that

$$\begin{array}{c} p_{j}\left(y;\theta \right)=\frac{\partial^{j}}{\partial \theta^{j}}\rho_{q}\left(y,F^{-1}\left(\theta \right)\right),\;j=0,1,\ldots , \end{array}$$

exist finitely up to any order required. For example, for j=1,

(6) $$\begin{array}{c} p_{1}\left(y;\theta \right)=\left\{\psi \left(r\left(y,\mu \right)\right)-G_{1}^{\prime }\left(\mu \right)\right\}\left\{q^{\prime \prime }\left(\mu \right)\sqrt{V(\mu )}\right\}/F^{\prime }\left(\mu \right), \end{array}$$

where $\mu =F^{-1}\left(\theta \right)$. Explicit expressions for $p_{j}\left(y;\theta \right)$ (j=2,3) can be found in Equation (3.7) of [1]. Then, the estimation equation for $\hat{\tilde{\beta }}$ is

$$\begin{array}{c} \frac{1}{n}\sum_{i=1}^{n}\psi_{\mathrm{RBD}}\left(Z_{ni};\tilde{\beta }\right)=0, \end{array}$$

where the score vector is

(7) $$\begin{array}{c} \psi_{\mathrm{RBD}}\left(z_{n};\tilde{\beta }\right)=p_{1}\left(y;\theta \right)w\left(x_{n}\right){\tilde{x}}_{n}, \end{array}$$

with $\theta ={\tilde{x}}_{n}^{T}\tilde{\beta }$. The consistency and asymptotic normality of $\hat{\tilde{\beta }}$ have been studied in [1]; see Theorems 1 and 2 therein.

Furthermore, to conduct statistical inference for the “general linear model”, the following hypotheses are considered,

(8) $$\begin{array}{c} H_{0}:A_{n}{\tilde{\beta }}_{n,0}=g_{0}\;\mathrm{versus}\;H_{1}:A_{n}{\tilde{\beta }}_{n,0}\neq g_{0}, \end{array}$$

where $A_{n}$ is a given $k\times (p_{n}+1)$ matrix such that $A_{n}A_{n}^{T}\to G$ with G being a k×k positive-definite matrix, and $g_{0}$ is a known k×1 vector.

To perform the test of Equation (8), Zhang et al. [1] proposed the Wald-type test statistic,

(9) $$\begin{array}{c} W_{n}=n{\left(A_{n}\hat{\tilde{\beta }}-g_{0}\right)}^{T}{\left(A_{n}{\hat{H}}_{n}^{-1}{\hat{\Omega }}_{n}{\hat{H}}_{n}^{-1}A_{n}^{T}\right)}^{-1}\left(A_{n}\hat{\tilde{\beta }}-g_{0}\right), \end{array}$$

constructed from the *robust-BD* estimator $\hat{\tilde{\beta }}$ in Equation (5), where

$$\begin{array}{ccc} {\hat{\Omega }}_{n} & = & \frac{1}{n}\sum_{i=1}^{n}p_{1}^{2}\left(Y_{i};{\tilde{X}}_{ni}^{T}\hat{\tilde{\beta }}\right)w^{2}\left(X_{ni}\right){\tilde{X}}_{ni}{\tilde{X}}_{ni}^{T},\\ {\hat{H}}_{n} & = & \frac{1}{n}\sum_{i=1}^{n}p_{2}\left(Y_{i};{\tilde{X}}_{ni}^{T}\hat{\tilde{\beta }}\right)w\left(X_{ni}\right){\tilde{X}}_{ni}{\tilde{X}}_{ni}^{T}. \end{array}$$

The asymptotic distributions of $W_{n}$ under the null and alternative hypotheses have been developed in [1]; see Theorems 4–6 therein.

On the other hand, the issue on the robustness of $W_{n}$, used for possibly contaminated data, remains unknown. Section 3 of this paper will address this issue with detailed derivations.

## 3. Robustness Properties of $W_{n}$ in Equation (9)

This section derives the influence function of the *robust-BD* Wald-type test and studies the influence of a small amount of contamination on the asymptotic level and power of the test. The proofs of the theoretical results are given in Appendix A.

Denote by $K_{n,0}$ the true distribution of $Z_{n}$ following the “general linear model” characterized by Equations (1) and (2). To facilitate the discussion of robustness properties, we consider the ϵ-contamination,

(10) $$\begin{array}{c} K_{n,\epsilon }=\left(1-\frac{\epsilon }{\sqrt{n}}\right)K_{n,0}+\frac{\epsilon }{\sqrt{n}}J, \end{array}$$

where *J* is an arbitrary distribution and ϵ>0 is a constant. Then, $K_{n,\epsilon }$ is a contaminated distribution of $Z_{n}$ with the amount of contamination converging to 0 at rate $1/\sqrt{n}$. Denote by $K_{n}$ the empirical distribution of ${\left\{{Z_{n}}_{i}\right\}}_{i=1}^{n}$.

For a generic distribution *K* of $Z_{n}$, define

(11) $$\begin{array}{ccc} \ell_{K}\left(\tilde{\beta }\right) & = & \;E_{K}\left\{\rho_{q}\left(Y,F^{-1}\left({\tilde{X}}_{n}^{T}\tilde{\beta }\right)\right)w\left(X_{n}\right)\right\},\\ S_{K} & = & \{\tilde{\beta }:\;E_{K}\left\{\psi_{\mathrm{RBD}}\left(Z_{n};\tilde{\beta }\right)\right\}=0\}, \end{array}$$

where $\rho_{q}\left(\cdot ,\cdot \right)$ and $\psi_{\mathrm{RBD}}\left(\cdot ;\cdot \right)$ are defined in Equations (4) and (7), respectively. It’s worth noting that the solution to $\;E_{K}\left\{\psi_{\mathrm{RBD}}\left(Z_{n};\tilde{\beta }\right)\right\}=0$ may not be unique, i.e., $S_{K}$ may contain more than one element. We then define a functional for the estimator of ${\tilde{\beta }}_{n,0}$ as follows,

(12) $$\begin{array}{c} T\left(K\right)=\underset{\tilde{\beta }\in S_{K}}{\arg\min}∥\tilde{\beta }-{\tilde{\beta }}_{n,0}∥. \end{array}$$

From the result of Lemma A1 in Appendix A, $T(K_{n,\epsilon })$ is the unique local minimizer of $\ell_{K_{n,\epsilon }}\left(\tilde{\beta }\right)$ in the $\sqrt{p_{n}/n}$-neighborhood of ${\tilde{\beta }}_{n,0}$. Particularly, $T\left(K_{n,0}\right)={\tilde{\beta }}_{n,0}$. Similarly, from Lemma A2 in Appendix A, $T(K_{n})$ is the unique local minimizer of $\ell_{K_{n}}\left(\tilde{\beta }\right)$ which satisfies $∥T\left(K_{n}\right)-{\tilde{\beta }}_{n,0}∥=O_{P}\left(\sqrt{p_{n}/n}\right)$.

From [23] (Equation (2.1.6) on pp. 84), the influence function of T(·) at $K_{n,0}$ is defined as

$$\begin{array}{c} \mathrm{IF}\left(z_{n};T,K_{n,0}\right)=\frac{\partial }{\partial t}T\left(\left(1-t\right)K_{n,0}+t\Delta_{z_{n}}\right){|}_{t=0}=\lim_{t↓0}\frac{T\left(\left(1-t\right)K_{n,0}+t\Delta_{z_{n}}\right)-{\tilde{\beta }}_{n,0}}{t}, \end{array}$$

where $\Delta_{z_{n}}$ is the probability measure which puts mass 1 at the point $z_{n}$. Since the dimension of T(·) diverges with *n*, its influence function is defined for each fixed *n*. From Lemma A8 in Appendix A, under certain regularity conditions, the influence function exists and has the following expression:

(13) $$\begin{array}{c} \mathrm{IF}\left(z_{n};T,K_{n,0}\right)=-H_{n}^{-1}\psi_{\mathrm{RBD}}\left(z_{n};{\tilde{\beta }}_{n,0}\right), \end{array}$$

where $H_{n}=\;E_{K_{n,0}}\left\{p_{2}\left(Y;{\tilde{X}}_{n}^{T}{\tilde{\beta }}_{n,0}\right)w\left(X_{n}\right){\tilde{X}}_{n}{\tilde{X}}_{n}^{T}\right\}$. The form of the influence function for diverging $p_{n}$ in Equation(13) coincides with that in [23,24] for fixed $p_{n}$.

In our theoretical derivations, approximations of the asymptotic level and power of $W_{n}$ will involve the following matrices:

$$\begin{array}{ccc} \Omega_{n} & = & \;E_{K_{n,0}}\left\{p_{1}^{2}\left(Y;{\tilde{X}}_{n}^{T}{\tilde{\beta }}_{n,0}\right)w^{2}\left(X_{n}\right){\tilde{X}}_{n}{\tilde{X}}_{n}^{T}\right\},\\ U_{n} & = & A_{n}H_{n}^{-1}\Omega_{n}H_{n}^{-1}A_{n}^{T}. \end{array}$$

### 3.1. Asymptotic Level of $W_{n}$ under Contamination

We now investigate the asymptotic level of the Wald-type test $W_{n}$ under the ϵ-contamination.

**Theorem** **1.** *Assume Conditions* A0–A9 *and* B4 *in Appendix A. Suppose $p_{n}^{6}/n\to 0$ as n→∞, $\sup_{n}\;E_{J}(∥w\left(X_{n}\right){\tilde{X}}_{n}∥)\leq C$. Denote by $\alpha (K_{n,\epsilon })$ the level of $W_{n}=n{\left\{A_{n}T\left(K_{n}\right)-g_{0}\right\}}^{T}{\left(A_{n}{\hat{H}}_{n}^{-1}{\hat{\Omega }}_{n}{\hat{H}}_{n}^{-1}A_{n}^{T}\right)}^{-1}\left\{A_{n}T\left(K_{n}\right)-g_{0}\right\}$ when the underlying distribution is $K_{n,\epsilon }$ in Equation* (10) *and by $\alpha_{{}_{0}}$ the nominal level. Under $H_{0}$ in Equation* (8), *it follows that*

$$\begin{array}{c} \underset{n\to \infty }{lim sup}\alpha \left(K_{n,\epsilon }\right)=\alpha_{{}_{0}}+\epsilon^{2}\mu_{k}D+o\left(\epsilon^{2}\right)\;\mathrm{as}\;\epsilon \to 0, \end{array}$$

*where*

$$\begin{array}{c} D=\underset{n\to \infty }{lim sup}{∥U_{n}^{-1/2}A_{n}\;E_{J}\left\{\mathrm{IF}\left(Z_{n};T,K_{n,0}\right)\right\}∥}^{2}<\infty , \end{array}$$

*$\mu_{k}=-\frac{\partial }{\partial \delta }H_{k}\left(\eta_{{}_{1-\alpha_{{}_{0}}}};\delta \right){|}_{\delta =0}$, $H_{k}\left(\cdot ;\delta \right)$ is the cumulative distribution function of a $\chi_{k}^{2}\left(\delta \right)$ distribution, and $\eta_{{}_{1-\alpha_{{}_{0}}}}$ is the $1-\alpha_{{}_{0}}$ quantile of the central $\chi_{k}^{2}$ distribution.*

Theorem 1 indicates that if the influence function for T(·) is bounded, then the asymptotic level of $W_{n}$ under the ϵ-contamination is also bounded and close to the nominal level when ϵ is sufficiently small. As a comparison, the robustness property in [10] of the Wald-type test is studied based on M-estimator for general parametric models with a fixed dimension $p_{n}$. They assumed certain conditions that guarantee Fréchet differentiability which further implies the existence of the influence function and the asymptotic normality of the corresponding estimator. However, in the set-ups of our paper, it’s difficult to check those conditions, due to the use of Bregman divergence and the diverging dimension $p_{n}$. Hence, the assumptions we make in Theorem 1 are different from those in [10], and are comparatively mild and easy to check. Moreover, the result of Theorem 1 cannot be easily derived from that of [10].

In Theorem 1, $p_{n}$ is allowed to diverge with $p_{n}^{6}/n=o\left(1\right)$, which is slower than that in [1] with $p_{n}^{5}/n=o\left(1\right)$. Theoretically, the assumption $p_{n}^{5}/n=o\left(1\right)$ is required to obtain the asymptotic distribution of $W_{n}$ in [1]. Furthermore, to derive the limit distribution of $W_{n}$ under the ϵ-contamination, assumption $p_{n}^{6}/n=o\left(1\right)$ is needed (see Lemma A7 in Appendix A). Hence, the reason that our assumption is stronger than that in [1] is the consideration of the ϵ-contamination of the data. Practically, due to the advancement of technology and different forms of data gathering, large dimension becomes a common characteristic and hence the varying-dimensional model has a wide range of applications, e.g., brain imaging data, financial data, web term-document data and gene expression data. Even some of the classical settings, e.g., the Framingham heart study with n=25,000 and $p_{n}=100$, can be viewed as varying-dimensional cases.

As an illustration, we apply the general result of Theorem 1 to the special case of a point mass contamination.

**Corollary** **1.** *With the notations in Theorem* 1, *assume Conditions* A0–A9 *in Appendix A,* $\sup_{x_{n}\in R^{p_{n}}}∥w\left(x_{n}\right)x_{n}∥\leq C$ *and* $\sup_{\mu \in R}\left|q^{\prime \prime }\left(\mu \right)\sqrt{V(\mu )}/F^{\prime }\left(\mu \right)\right|\leq C$.

(i) *If $p_{n}\equiv p$, $A_{n}\equiv A$, ${\tilde{\beta }}_{n,0}\equiv {\tilde{\beta }}_{0}$, $K_{n,0}\equiv K_{0}$ and $U_{n}\equiv U$ are fixed, then, for $K_{n,\epsilon }=\left(1-\epsilon /\sqrt{n}\right)K_{0}+\epsilon /\sqrt{n}\Delta_{z}$ with $z\in R^{p}$ a fixed point, under $H_{0}$ in Equation* (8), *it follows that*
  $$\begin{array}{c} \underset{z\in R^{p}}{\sup}\lim_{n\to \infty }\alpha \left(K_{n,\epsilon }\right)=\alpha_{{}_{0}}+\epsilon^{2}\mu_{k}D_{1}+o\left(\epsilon^{2}\right)\;\mathrm{as}\;\epsilon \to 0, \end{array}$$
  *where*
  $$\begin{array}{c} D_{1}=\underset{z\in R^{p}}{\sup}{∥U^{-1/2}A\;\mathrm{IF}\left(z;T,K_{0}\right)∥}^{2}<\infty . \end{array}$$
(ii) *If $p_{n}$ diverges with $p_{n}^{6}/n\to 0$, for $K_{n,\epsilon }=\left(1-\epsilon /\sqrt{n}\right)K_{n,0}+\epsilon /\sqrt{n}\Delta_{z_{n}}$ with $z_{n}\in R^{p_{n}}$ a sequence of deterministic points, then, under $H_{0}$ in Equation* (8),
  $$\begin{array}{c} \underset{C_{0}>0}{\sup}\underset{z_{n}\in S_{C_{0}}}{\sup}\underset{n\to \infty }{lim sup}\alpha \left(K_{n,\epsilon }\right)=\alpha_{{}_{0}}+\epsilon^{2}\mu_{k}D_{2}+o\left(\epsilon^{2}\right)\;\mathrm{as}\;\epsilon \to 0, \end{array}$$
  *where $S_{C_{0}}=\{z_{n}={\left(x_{n}^{T},y\right)}^{T}:∥x_{n}{∥}_{\infty }\leq C_{0}\}$, $C_{0}>0$ is a constant and*
  $$\begin{array}{c} D_{2}=\underset{C_{0}>0}{\sup}\underset{z_{n}\in S_{C_{0}}}{\sup}\underset{n\to \infty }{lim sup}{∥U_{n}^{-1/2}A_{n}\mathrm{IF}\left(z_{n};T,K_{n,0}\right)∥}^{2}<\infty . \end{array}$$

In Corollary 1, conditions $\sup_{x_{n}\in R^{p_{n}}}∥w\left(x_{n}\right)x_{n}∥\leq C$ and $\sup_{\mu \in R}\left|q^{\prime \prime }\left(\mu \right)\sqrt{V(\mu )}/F^{\prime }\left(\mu \right)\right|\leq C$ are needed to guarantee the boundedness of the score function in Equation (7). Particularly, the function $w(x_{n})$ downweights the high leverage points and can be chosen as, e.g., $w\left(x_{n}\right)=1/(1+∥x_{n}∥)$. The condition $\sup_{\mu \in R}\left|q^{\prime \prime }\left(\mu \right)\sqrt{V(\mu )}/F^{\prime }\left(\mu \right)\right|\leq C$ is needed to bound Equation (6), and is satisfied in many situations.

- For example, for the linear model with $q\left(\mu \right)=a\mu -\mu^{2}$, $V\left(\mu \right)=\sigma^{2}$ and F(μ)=μ, where *a* and $\sigma^{2}$ are constants, we observe $|q^{\prime \prime }\left(\mu \right)\sqrt{V(\mu )}/F^{\prime }\left(\mu \right)|=2\sigma \leq C$.
- Another example is the logistic regression model with binary response and q(μ)=−2{μlog(μ)+(1−μ)log(1−μ)} (corresponding to Bernoulli deviance loss), V(μ)=μ(1−μ), F(μ)=log{μ/(1−μ)}. In this case, $|q^{\prime \prime }\left(\mu \right)\sqrt{V(\mu )}/F^{\prime }{\left(\mu \right)|=2\left\{\mu \left(1-\mu \right)\right\}}^{1/2}\leq C$ since μ∈[0,1]. Likewise, if $q\left(\mu \right)=2{\left\{\mu \left(1-\mu \right)\right\}}^{1/2}$ (for the exponential loss), then $|q^{\prime \prime }\left(\mu \right)\sqrt{V(\mu )}/F^{\prime }\left(\mu \right)|=1/2$.

Furthermore, the bound on ψ(·) is useful to control deviations in the *Y*-space, which ensures the stability of the *robust-BD* test if *Y* is arbitrarily contaminated.

Concerning the dimensionality $p_{n}$, Corollary 1 reveals the following implications. If $p_{n}$ is fixed, then the asymptotic level of $W_{n}$ under the ϵ-contamination is uniformly bounded for all $z\in R^{p}$, which implies the robustness of validity of the test. This result coincides with that in Proposition 5 of [10]. When $p_{n}$ diverges, the asymptotic level is still stable if the point contamination satisfies $∥x_{n}{∥}_{\infty }\leq C_{0}$, where $C_{0}>0$ is an arbitrary constant. Although this condition may not be the weakest, it still covers a wide range of point mass contaminations.

### 3.2. Asymptotic Power of $W_{n}$ under Contamination

Now, we will study the asymptotic power of $W_{n}$ under a sequence of contiguous alternatives of the form

(14) $$\begin{array}{c} H_{1n}:A_{n}{\tilde{\beta }}_{n,0}-g_{0}=n^{-1/2}c, \end{array}$$

where $c={\left(c_{1},\ldots ,c_{k}\right)}^{T}\neq 0$ is fixed.

**Theorem** **2.** *Assume Conditions A0–A9 and B4 in Appendix A. Suppose $p_{n}^{6}/n\to 0$ as n→∞, $\sup_{n}\;E_{J}(∥w\left(X_{n}\right){\tilde{X}}_{n}∥)\leq C$. Denote by $\beta (K_{n,\epsilon })$ the power of $W_{n}=n{\left\{A_{n}T\left(K_{n}\right)-g_{0}\right\}}^{T}{\left(A_{n}{\hat{H}}_{n}^{-1}{\hat{\Omega }}_{n}{\hat{H}}_{n}^{-1}A_{n}^{T}\right)}^{-1}\left\{A_{n}T\left(K_{n}\right)-g_{0}\right\}$ when the underlying distribution is $K_{n,\epsilon }$ in Equation* (10) *and by $\beta_{0}$ the nominal power. Under $H_{1n}$ in Equation* (14), *it follows that*

$$\begin{array}{c} \underset{n\to \infty }{lim inf}\beta \left(K_{n,\epsilon }\right)=\beta_{0}+\epsilon \nu_{k}B+o\left(\epsilon \right)\;\mathrm{as}\;\epsilon \to 0, \end{array}$$

*where*

$$\begin{array}{c} B=\underset{n\to \infty }{lim inf}2c^{T}U_{n}^{-1}A_{n}\;E_{J}\left\{\mathrm{IF}\left(Z_{n};T,K_{n,0}\right)\right\}, \end{array}$$

*with |B|<∞, $\nu_{k}=-\frac{\partial }{\partial \delta }H_{k}\left(\eta_{{}_{1-\alpha_{{}_{0}}}};\delta \right){|}_{\delta =c^{T}U_{n}^{-1}c}$ and $H_{k}\left(\cdot ;\delta \right)$ and $\eta_{{}_{1-\alpha_{{}_{0}}}}$ being defined in Theorem* 1.

The result for the asymptotic power is similar in spirit to that for the level. From Theorem 2, if the influence function is bounded, the asymptotic power is also bounded from below and close to the nominal power under a small amount of contamination. This means that the *robust-BD* Wald-type test enjoys the robustness of efficiency. In addition, the property of the asymptotic power can be obtained for a point mass contamination.

**Corollary** **2.** *With the notations in Theorem* 2, *assume Conditions* A0–A9 *in Appendix A,* $\sup_{x_{n}\in R^{p_{n}}}∥w\left(x_{n}\right)x_{n}∥\leq C$ *and* $\sup_{\mu \in R}\left|q^{\prime \prime }\left(\mu \right)\sqrt{V(\mu )}/F^{\prime }\left(\mu \right)\right|\leq C$.

(i) *If* $p_{n}\equiv p$, $A_{n}\equiv A$, ${\tilde{\beta }}_{n,0}\equiv {\tilde{\beta }}_{0}$, $K_{n,0}\equiv K_{0}$ *and* $U_{n}\equiv U$ *are fixed, then, for* $K_{n,\epsilon }=\left(1-\epsilon /\sqrt{n}\right)K_{0}+\epsilon /\sqrt{n}\Delta_{z}$ *with* $z\in R^{p}$ *a fixed point, under* $H_{1n}$ *in Equation* (14), *it follows that*
  $$\begin{array}{c} \underset{z\in R^{p}}{\inf}\lim_{n\to \infty }\beta \left(K_{n,\epsilon }\right)=\beta_{0}+\epsilon \nu_{k}B_{1}+o\left(\epsilon \right)\;\mathrm{as}\;\epsilon \to 0, \end{array}$$
  *where*
  $$\begin{array}{c} B_{1}=\underset{z\in R^{p}}{\inf}2c^{T}U^{-1}A\;\mathrm{IF}\left(z;T,K_{0}\right), \end{array}$$
  *with* $|B_{1}|<\infty$.
(ii) *If* $p_{n}$ *diverges with* $p_{n}^{6}/n\to 0$, *for* $K_{n,\epsilon }=\left(1-\epsilon /\sqrt{n}\right)K_{n,0}+\epsilon /\sqrt{n}\Delta_{z_{n}}$ *with* $z_{n}\in R^{p_{n}}$ *a sequence of deterministic points, then, under* $H_{1n}$ *in Equation* (14),
  $$\begin{array}{c} \underset{C_{0}>0}{\inf}\underset{z_{n}\in S_{C_{0}}}{\inf}\underset{n\to \infty }{lim inf}\beta \left(K_{n,\epsilon }\right)=\beta_{0}+\epsilon \nu_{k}B_{2}+o\left(\epsilon \right)\;\mathrm{as}\;\epsilon \to 0, \end{array}$$
  *where* $S_{C_{0}}=\{z_{n}={\left(x_{n}^{T},y\right)}^{T}:∥x_{n}{∥}_{\infty }\leq C_{0}\}$, $C_{0}>0$ *is a constant and*
  $$\begin{array}{c} B_{2}=\underset{C_{0}>0}{\inf}\underset{z_{n}\in S_{C_{0}}}{\inf}\underset{n\to \infty }{lim inf}2c^{T}U_{n}^{-1}A_{n}\mathrm{IF}\left(Z_{n};T,K_{n,0}\right), \end{array}$$
  *with* $|B_{2}|<\infty$.

## 4. Simulation

Regarding the practical utility of $W_{n}$, numerical studies concerning the empirical level and power of $W_{n}$ under a fixed amount of contamination have been conducted in Section 6 of [1]. To support the theoretical results in our paper, we conduct new simulations to check the robustness of validity and efficiency of $W_{n}$. Specifically, we will examine the empirical level and power of the test statistic as ϵ varies.

The *robust-BD* estimation utilizes the Huber ψ-function $\psi_{c}\left(\cdot \right)$ with c=1.345 and the weight function $w\left(X_{n}\right)=1/(1+∥X_{n}∥)$. Comparisons are made with the classical non-robust counterparts corresponding to using ψ(r)=r and $w(x_{n})\equiv 1$. For each situation below, we set n=1000 and conduct 400 replications.

### 4.1. Overdispersed Poisson Responses

Overdispersed Poisson counts *Y*, satisfying $\mathrm{var}\left(Y|X_{n}=x_{n}\right)=2m\left(x_{n}\right)$, are generated via a negative Binomial $\left(m\left(x_{n}\right),1/2\right)$ distribution. Let $p_{n}=\left\lfloor 4\left(n^{1/5.5}-1\right)\right\rfloor$ and ${\tilde{\beta }}_{n,0}={\left(0,2,0,\ldots ,0\right)}^{T}$, where ⌊·⌋ denotes the floor function. Generate $X_{ni}={\left(X_{i,1},\ldots ,X_{i,p_{n}}\right)}^{T}$ by $X_{i,j}\overset{i.i.d.}{∼}\mathrm{Unif}\left[-0.5,\;0.5\right]$. The log link function is considered and the (negative) quasi-likelihood is utilized as the BD, generated by the *q*-function in Equation (3) with V(μ)=μ. The estimator and test statistic are calculated by assuming *Y* follows Poisson distribution.

The data are contaminated by $X_{i,\mathrm{mod}(i,p_{n}-1)+1}^{*}=3\mathrm{sign}\left(U_{i}-0.5\right)$ and $Y_{i}^{*}=Y_{i}I\left(Y_{i}>20\right)+20I\left(Y_{i}\leq 20\right)$ for i=1,…,k, with k∈{2,4,6,8,10,12,14,16} the number of contaminated data points, where mod(a,b) is the modulo operation “*a* modulo *b*” and $\left\{U_{i}\right\}\overset{i.i.d.}{∼}\mathrm{Unif}\left(0,1\right)$. Then, the proportion of contaminated data, k/n, is equal to $\epsilon /\sqrt{n}$ as in Equation (10), which implies $\epsilon =k/\sqrt{n}$.

**Figure 1:**
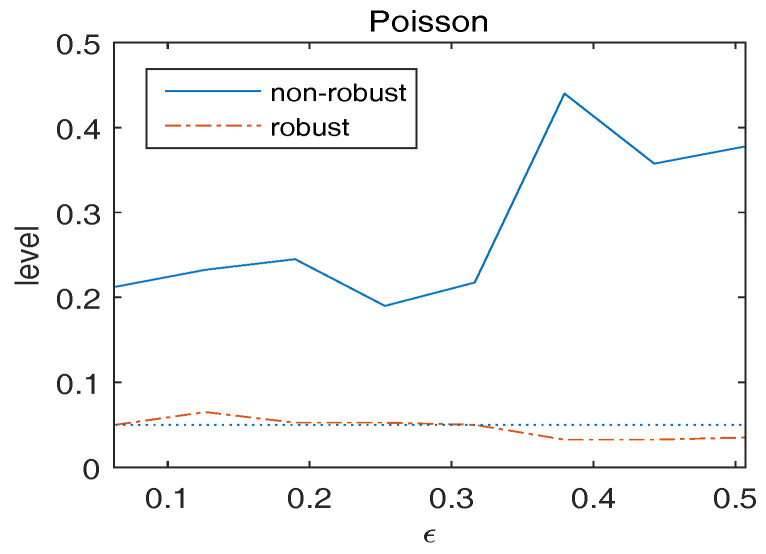
Observed level of $W_{n}$ versus ϵ for overdispersed Poisson responses. The dotted line indicates the 5% significance level.

Consider the null hypothesis $H_{0}:A_{n}{\tilde{\beta }}_{n,0}=0$ with $A_{n}=\left(0,0,0,1,0,\ldots ,0\right)$. Figure 1 plots the empirical level of $W_{n}$ versus ϵ. We observe that the asymptotic nominal level 0.05 is approximately retained by the robust Wald-type test. On the other hand, under contaminations, the non-robust Wald-type test breaks in level, showing high sensitivity to the presence of outliers.

**Figure 2:**
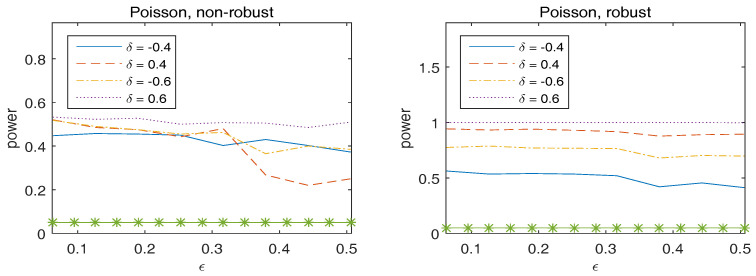
Observed power of $W_{n}$ versus ϵ for overdispersed Poisson responses. The statistics in the left panel correspond to non-robust method and those in the right panel are for robust method. The asterisk line indicates the 5% significance level.

To assess the stability of the power of the test, we generate the original data from the true model, but with the true parameter ${\tilde{\beta }}_{n,0}$ replaced by ${\tilde{\beta }}_{n}={\tilde{\beta }}_{n,0}+\delta c$ with δ∈{−0.4,0.4,−0.6,0.6} and $c={\left(1,\ldots ,1\right)}^{T}$ a vector of ones. Figure 2 plots the empirical rejection rates of the null model, which implies that the robust Wald-type test has sufficiently large power to detect the alternative hypothesis. In addition, the power of the robust method is generally larger than that of the non-robust method.

### 4.2. Bernoulli Responses

**Figure 3:**
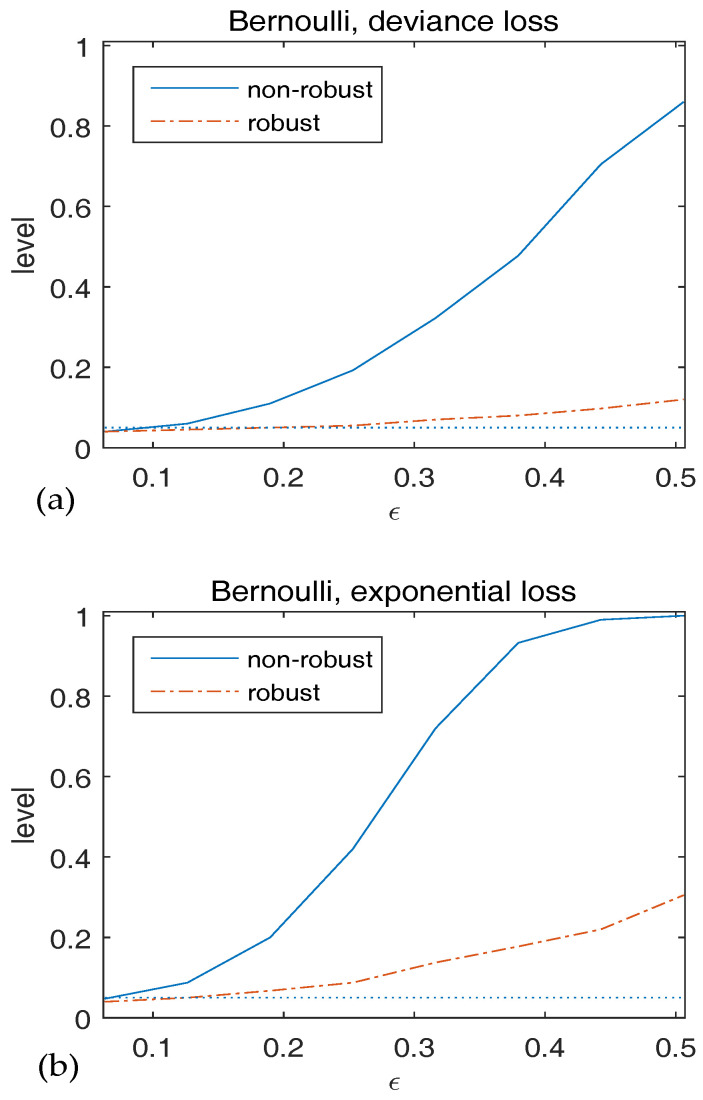
Observed level of $W_{n}$ versus ϵ for Bernoulli responses. The statistics in (**a**) use deviance loss and those in (**b**) use exponential loss. The dotted line indicates the 5% significancelevel.

We generate data with two classes from the model, $Y|X_{n}=x_{n}∼\mathrm{Bernoulli}\left\{m\left(x_{n}\right)\right\}$, where $\mathrm{logit}\left\{m\left(x_{n}\right)\right\}={\tilde{x}}_{n}^{T}{\tilde{\beta }}_{n,0}$. Let $p_{n}=2$, ${\tilde{\beta }}_{n,0}={\left(0,1,1\right)}^{T}$ and $X_{ni}\overset{i.i.d.}{∼}N\left(0,I_{p_{n}}\right)$. The null hypothesis is $H_{0}:{\tilde{\beta }}_{n,0}={\left(0,1,1\right)}^{T}$. Both the deviance loss and the exponential loss are employed as the BD. We contaminate the data by setting $X_{i,1}^{*}=2+i/8$ and $Y_{i}^{*}=0$ for i=1,…,k with k∈{2,4,6,8,10,12,14,16}. To investigate the robustness of validity of $W_{n}$, we plot the observed level versus ϵ in Figure 3. We find that the level of the non-robust method diverges fast as ϵ increases. It’s also clear that the empirical level of the robust method is close to the nominal level when ϵ is small and increases slightly with ϵ, which coincides with our results in Theorem 1.

**Figure 4:**
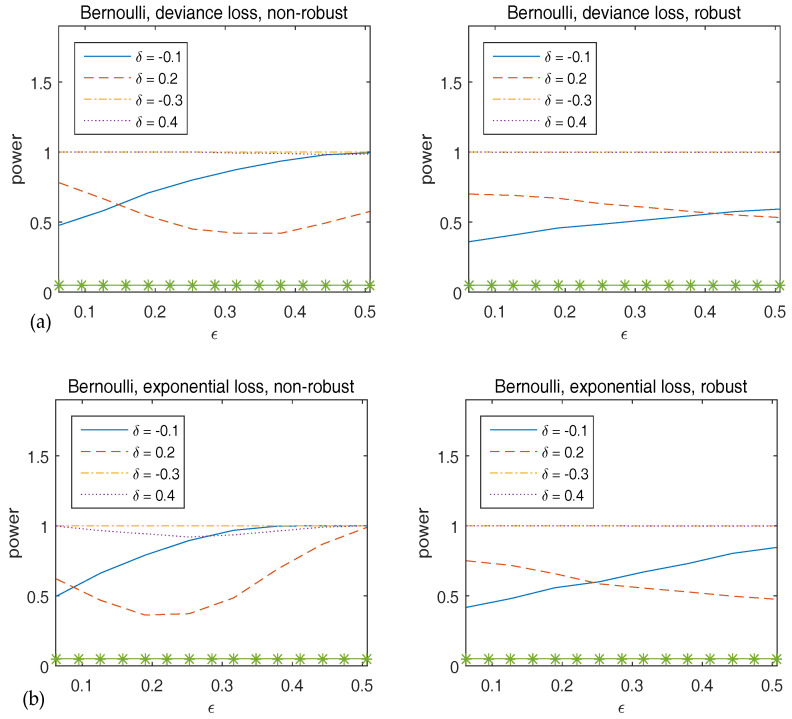
Observed power of $W_{n}$ versus ϵ for Bernoulli responses. The top panels correspond to deviance loss while the bottom panels are for exponential loss. The statistics in the left panels are calculated using non-robust method and those in the right panels are from robust method. The asterisk line indicates the 5% significance level.

To assess the stability of the power of $W_{n}$, we generate the original data from the true model, but with the true parameter ${\tilde{\beta }}_{n,0}$ replaced by ${\tilde{\beta }}_{n}={\tilde{\beta }}_{n,0}+\delta c$ with δ∈{−0.1,0.2,−0.3,0.4} and $c={\left(1,\ldots ,1\right)}^{T}$ a vector of ones. Figure 4 plots the power of the Wald-type test versus ϵ, which implies that the robust method has sufficiently large power, and hence supports the theoretical results in Theorem 2.

## Appendix A. Conditions and Proofs of Main Results

We first introduce some necessary notations used in the proof.

**Notations.** For arbitrary distributions *K* and $K^{\prime }$ of $Z_{n}$, define

$$\begin{array}{ccc} \Omega_{n,K,T(K^{\prime })} & = & \;E_{K}\left\{p_{1}^{2}\left(Y;{\tilde{X}}_{n}^{T}T\left(K^{\prime }\right)\right)w^{2}\left(X_{n}\right){\tilde{X}}_{n}{\tilde{X}}_{n}^{T}\right\},\\ H_{n,K,T(K^{\prime })} & = & \;E_{K}\left\{p_{2}\left(Y;{\tilde{X}}_{n}^{T}T\left(K^{\prime }\right)\right)w\left(X_{n}\right){\tilde{X}}_{n}{\tilde{X}}_{n}^{T}\right\}. \end{array}$$

Therefore, $\Omega_{n}=\Omega_{n,K_{n,0},{\tilde{\beta }}_{n,0}}$, $H_{n}=H_{n,K_{n,0},{\tilde{\beta }}_{n,0}}$, ${\hat{\Omega }}_{n}=\Omega_{n,K_{n},T\left(K_{n}\right)}$ and ${\hat{H}}_{n}=H_{n,K_{n},T\left(K_{n}\right)}$. For notational simplicity, let $\Omega_{n,\epsilon }=\Omega_{n,K_{n,\epsilon },T\left(K_{n,\epsilon }\right)}$ and $H_{n,\epsilon }=H_{n,K_{n,\epsilon },T\left(K_{n,\epsilon }\right)}$.

Define the following matrices,

$$\begin{array}{ccc} U(K_{n,\epsilon }) & = & A_{n}H_{n,\epsilon }^{-1}\Omega_{n,\epsilon }H_{n,\epsilon }^{-1}A_{n}^{T},\;\\ U(K_{n}) & = & A_{n}{\hat{H}}_{n}^{-1}{\hat{\Omega }}_{n}{\hat{H}}_{n}^{-1}A_{n}^{T}. \end{array}$$

The following conditions are needed in the proof, which are adopted from [1].

**Condition A.**

A0. $\sup_{n\geq 1}{∥{\tilde{\beta }}_{n,0}∥}_{1}<\infty$.
A1. w(·) is a bounded function. Assume that ψ(r) is a bounded, odd function, and twice differentiable, such that $\psi^{\prime }\left(r\right)$, $\psi^{\prime }\left(r\right)r$, $\psi^{\prime \prime }\left(r\right)$, $\psi^{\prime \prime }\left(r\right)r$ and $\psi^{\prime \prime }\left(r\right)r^{2}$ are bounded; V(·)>0, $V^{\left(2\right)}$ is continuous.
A2. $q^{\left(4\right)}\left(\cdot \right)$ is continuous, and $q^{\left(2\right)}\left(\cdot \right)<0$. $G_{1}^{\left(3\right)}$ is continuous.
A3. F(·) is monotone and a bijection, $F^{\left(3\right)}\left(\cdot \right)$ is continuous, and $F^{\left(1\right)}\left(\cdot \right)\neq 0$.
A4. $∥X_{n}{∥}_{\infty }\leq C$ almost surely if the underlying distribution is $K_{n,0}$.
A5. $\;E_{K_{n,0}}\left({\tilde{X}}_{n}{\tilde{X}}_{n}^{T}\right)$ exists and is nonsingular.
A6. There is a large enough open subset of $R^{p_{n}+1}$ which contains ${\tilde{\beta }}_{n,0}$, such that $F^{-1}\left({\tilde{x}}_{n}^{T}\tilde{\beta }\right)$ is bounded for all $\tilde{\beta }$ in the subset and all ${\tilde{x}}_{n}$ such that $∥{\tilde{x}}_{n}{∥}_{\infty }\leq C$, where C>0 is a large enough constant.
A7. $H_{n}$ is positive definite, with eigenvalues uniformly bounded away from 0.
A8. $\Omega_{n}$ is positive definite, with eigenvalues uniformly bounded away from 0.
A9. $∥H_{n}^{-1}\Omega_{n}∥$ is bounded away from *∞*.

**Condition B.**

B4. $∥X_{n}{∥}_{\infty }\leq C$ almost surely if the underlying distribution is *J*.

The following Lemmas A1–A9 are needed to prove the main theoretical results in this paper.

**Lemma A1** ($∥T\left(K_{n,\epsilon }\right)-{\tilde{\beta }}_{n,0}∥$)**.** *Assume Conditions* A0–A7 *and* B4. *For $K_{n,\epsilon }$ in Equation* (10), $\ell_{K}\left(\cdot \right)$ *in Equation* (11) *and T(·) in Equation* (12), *if $p_{n}^{4}/n\to 0$ as n→∞, then $T(K_{n,\epsilon })$ is a local minimizer of $\ell_{K_{n,\epsilon }}\left(\tilde{\beta }\right)$ such that $∥T\left(K_{n,\epsilon }\right)-{\tilde{\beta }}_{n,0}∥=O\left(\sqrt{p_{n}/n}\right)$. Furthermore, $T(K_{n,\epsilon })$ is unique.*

**Proof.** We follow the idea of the proof in [25]. Let $r_{n}=\sqrt{p_{n}/n}$ and ${\tilde{u}}_{n}={\left(u_{0},u_{1},\ldots ,u_{p_{n}}\right)}^{T}\in R^{p_{n}+1}$. First, we show that there exists a sufficiently large constant *C* such that, for large *n*, we have

(A1) $$\begin{array}{c} \underset{∥{\tilde{u}}_{n}∥=C}{\inf}\ell_{K_{n,\epsilon }}\left({\tilde{\beta }}_{n,0}+r_{n}{\tilde{u}}_{n}\right)>\ell_{K_{n,\epsilon }}\left({\tilde{\beta }}_{n,0}\right). \end{array}$$

To show Equation (A1), consider

$$\begin{array}{ccc} \ell_{K_{n,\epsilon }}\left({\tilde{\beta }}_{n,0}+r_{n}{\tilde{u}}_{n}\right)-\ell_{K_{n,\epsilon }}\left({\tilde{\beta }}_{n,0}\right) & = & \;E_{K_{n,\epsilon }}\{\rho_{q}(Y,F^{-1}\left({\tilde{X}}_{n}^{T}{\tilde{\beta }}_{n,0}+r_{n}{\tilde{X}}_{n}^{T}{\tilde{u}}_{n}\right))w\left(X_{n}\right)\\ & & -\rho_{q}\left(Y,F^{-1}\left({\tilde{X}}_{n}^{T}{\tilde{\beta }}_{n,0}\right)\right)w\left(X_{n}\right)\}\\ & \equiv & I_{1}, \end{array}$$

where $∥{\tilde{u}}_{n}∥=C$. By Taylor expansion,

(A2) $$\begin{array}{c} I_{1}=I_{1,1}+I_{1,2}+I_{1,3}, \end{array}$$

where

$$\begin{array}{ccc} I_{1,1} & = & r_{n}\;E_{K_{n,\epsilon }}\left\{p_{1}\left(Y;{\tilde{X}}_{n}^{T}{\tilde{\beta }}_{n,0}\right)w\left(X_{n}\right){\tilde{X}}_{n}^{T}\right\}{\tilde{u}}_{n},\\ I_{1,2} & = & r_{n}^{2}/2\;E_{K_{n,\epsilon }}\left\{p_{2}\left(Y;{\tilde{X}}_{n}^{T}{\tilde{\beta }}_{n,0}\right)w\left(X_{n}\right){\left({\tilde{X}}_{n}^{T}{\tilde{u}}_{n}\right)}^{2}\right\},\\ I_{1,3} & = & r_{n}^{3}/6\;E_{K_{n,\epsilon }}\left\{p_{3}\left(Y;{\tilde{X}}_{n}^{T}{\tilde{\beta }}_{n}^{*}\right)w\left(X_{n}\right){\left({\tilde{X}}_{n}^{T}{\tilde{u}}_{n}\right)}^{3}\right\}, \end{array}$$

for ${\tilde{\beta }}_{n}^{*}$ located between ${\tilde{\beta }}_{n,0}$ and ${\tilde{\beta }}_{n,0}+r_{n}{\tilde{u}}_{n}$. Hence

$$\begin{array}{ccc} |I_{1,1}| & \leq & r_{n}∥\;E_{K_{n,\epsilon }}\left\{p_{1}\left(Y;{\tilde{X}}_{n}^{T}{\tilde{\beta }}_{n,0}\right)w\left(X_{n}\right){\tilde{X}}_{n}\right\}∥∥{\tilde{u}}_{n}∥\\ & = & r_{n}\frac{\epsilon }{\sqrt{n}}∥\;E_{J}\left\{p_{1}\left(Y;{\tilde{X}}_{n}^{T}{\tilde{\beta }}_{n,0}\right)w\left(X_{n}\right){\tilde{X}}_{n}\right\}∥∥{\tilde{u}}_{n}∥\\ & \leq & Cr_{n}\sqrt{p_{n}/n}∥{\tilde{u}}_{n}∥, \end{array}$$

since $∥\;E_{J}\left\{p_{1}\left(Y;{\tilde{X}}_{n}^{T}{\tilde{\beta }}_{n,0}\right)w\left(X_{n}\right){\tilde{X}}_{n}\right\}∥=O\left(\sqrt{p_{n}}\right)$ and $\;E_{K_{n,0}}\left\{p_{1}\left(Y;{\tilde{X}}_{n}^{T}{\tilde{\beta }}_{n,0}\right)w\left(X_{n}\right){\tilde{X}}_{n}\right\}=0$. For $I_{1,2}$ in Equation (A2),

$$\begin{array}{ccc} I_{1,2} & = & \frac{r_{n}^{2}}{2}\;E_{K_{n,0}}\left\{p_{2}\left(Y;{\tilde{X}}_{n}^{T}{\tilde{\beta }}_{n,0}\right)w\left(X_{n}\right){\left({\tilde{X}}_{n}^{T}{\tilde{u}}_{n}\right)}^{2}\right\}\\ & & +\frac{r_{n}^{2}}{2}\left[\;E_{K_{n,\epsilon }}\left\{p_{2}\left(Y;{\tilde{X}}_{n}^{T}{\tilde{\beta }}_{n,0}\right)w\left(X_{n}\right){\left({\tilde{X}}_{n}^{T}{\tilde{u}}_{n}\right)}^{2}\right\}-\;E_{K_{n,0}}\left\{p_{2}\left(Y;{\tilde{X}}_{n}^{T}{\tilde{\beta }}_{n,0}\right)w\left(X_{n}\right){\left({\tilde{X}}_{n}^{T}{\tilde{u}}_{n}\right)}^{2}\right\}\right]\\ & \equiv & I_{1,2,1}+I_{1,2,2}, \end{array}$$

where $I_{1,2,1}=2^{-1}r_{n}^{2}{\tilde{u}}_{n}^{T}H_{n}{\tilde{u}}_{n}$. Meanwhile, we have

$$\begin{array}{cc} & |I_{1,2,2}|\\ \leq & r_{n}^{2}∥\;E_{K_{n,\epsilon }}\left\{p_{2}\left(Y;{\tilde{X}}_{n}^{T}{\tilde{\beta }}_{n,0}\right)w\left(X_{n}\right){\tilde{X}}_{n}{\tilde{X}}_{n}^{T}\right\}-\;E_{K_{n,0}}\left\{p_{2}\left(Y;{\tilde{X}}_{n}^{T}{\tilde{\beta }}_{n,0}\right)w\left(X_{n}\right){\tilde{X}}_{n}{\tilde{X}}_{n}^{T}\right\}{∥}_{F}{∥{\tilde{u}}_{n}∥}^{2}\\ = & r_{n}^{2}\frac{\epsilon }{\sqrt{n}}∥\;E_{J}\left\{p_{2}\left(Y;{\tilde{X}}_{n}^{T}{\tilde{\beta }}_{n,0}\right)w\left(X_{n}\right){\tilde{X}}_{n}{\tilde{X}}_{n}^{T}\right\}-\;E_{K_{n,0}}\left\{p_{2}\left(Y;{\tilde{X}}_{n}^{T}{\tilde{\beta }}_{n,0}\right)w\left(X_{n}\right){\tilde{X}}_{n}{\tilde{X}}_{n}^{T}\right\}{∥}_{F}{∥{\tilde{u}}_{n}∥}^{2}\\ \leq & Cr_{n}^{2}p_{n}{∥{\tilde{u}}_{n}∥}^{2}/\sqrt{n}, \end{array}$$

where $∥\;E_{J}\left\{p_{2}\left(Y;{\tilde{X}}_{n}^{T}{\tilde{\beta }}_{n,0}\right)w\left(X_{n}\right){\tilde{X}}_{n}{\tilde{X}}_{n}^{T}\right\}{∥}_{F}=O\left(p_{n}\right)$ and $∥\;E_{K_{n,0}}\left\{p_{2}\left(Y;{\tilde{X}}_{n}^{T}{\tilde{\beta }}_{n,0}\right)w\left(X_{n}\right){\tilde{X}}_{n}{\tilde{X}}_{n}^{T}\right\}{∥}_{F}=O\left(p_{n}\right)$. Thus,

(A3) $$\begin{array}{c} I_{1,2}=2^{-1}r_{n}^{2}{\tilde{u}}_{n}^{T}H_{n}{\tilde{u}}_{n}+O\left(r_{n}^{2}p_{n}/\sqrt{n}\right){∥{\tilde{u}}_{n}∥}^{2}. \end{array}$$

For $I_{1,3}$ in Equation (A2), we observe that

$$\begin{array}{c} |I_{1,3}|\leq Cr_{n}^{3}\;E_{K_{n,\epsilon }}\left\{\right|p_{3}\left(Y;{\tilde{X}}_{n}^{T}{\tilde{\beta }}_{n}^{*}\right)|w\left(X_{n}\right)|{\tilde{X}}_{n}^{T}{\tilde{u}}_{n}{|}^{3}\}=O\left(r_{n}^{3}p_{n}^{3/2}\right){∥{\tilde{u}}_{n}∥}^{3}. \end{array}$$

We can choose some large *C* such that $I_{1,1}$ , $I_{1,2,2}$ and $I_{1,3}$ are all dominated by the first term of $I_{1,2}$ in Equation (A3), which is positive by the eigenvalue assumption. This implies Equation (A1). Therefore, there exists a local minimizer of $\ell_{K_{n,\epsilon }}\left(\tilde{\beta }\right)$ in the $\sqrt{p_{n}/n}$ neighborhood of ${\tilde{\beta }}_{n,0}$, and denote this minimizer by ${\tilde{\beta }}_{n,\epsilon }$. Next, we show that the local minimizer ${\tilde{\beta }}_{n,\epsilon }$ of $\ell_{K_{n,\epsilon }}\left(\tilde{\beta }\right)$ is unique in the $\sqrt{p_{n}/n}$ neighborhood of ${\tilde{\beta }}_{n,0}$. For all $\tilde{\beta }$ such that $∥\tilde{\beta }-{\tilde{\beta }}_{n,0}∥=O\left(n^{-1/4}p_{n}^{-1/2}\right)$,

$$\begin{array}{ccc} \;E_{K_{n,\epsilon }}∥\frac{\partial }{\partial \tilde{\beta }}\rho_{q}\left(Y,F^{-1}\left({\tilde{X}}_{n}^{T}\tilde{\beta }\right)\right)w\left(X_{n}\right)∥ & = & \;E_{K_{n,\epsilon }}∥p_{1}\left(Y;{\tilde{X}}_{n}^{T}\tilde{\beta }\right)w\left(X_{n}\right){\tilde{X}}_{n}∥\leq C\sqrt{p_{n}}\\ \;E_{K_{n,\epsilon }}∥\frac{\partial^{2}}{\partial {\tilde{\beta }}^{2}}\rho_{q}\left(Y,F^{-1}\left({\tilde{X}}_{n}^{T}\tilde{\beta }\right)\right)w\left(X_{n}\right)∥ & = & \;E_{K_{n,\epsilon }}∥p_{2}\left(Y;{\tilde{X}}_{n}^{T}\tilde{\beta }\right)w\left(X_{n}\right){\tilde{X}}_{n}X_{n}^{T}∥\leq Cp_{n} \end{array}$$

and hence,

$$\begin{array}{ccc} \frac{\partial }{\partial \tilde{\beta }}\;E_{K_{n,\epsilon }}\left\{\rho_{q}\left(Y,F^{-1}\left({\tilde{X}}_{n}^{T}\tilde{\beta }\right)\right)w\left(X_{n}\right)\right\} & = & \;E_{K_{n,\epsilon }}\left\{\frac{\partial }{\partial \tilde{\beta }}\rho_{q}\left(Y,F^{-1}\left({\tilde{X}}_{n}^{T}\tilde{\beta }\right)\right)w\left(X_{n}\right)\right\}\\ \frac{\partial^{2}}{\partial {\tilde{\beta }}^{2}}\;E_{K_{n,\epsilon }}\left\{\rho_{q}\left(Y,F^{-1}\left({\tilde{X}}_{n}^{T}\tilde{\beta }\right)\right)w\left(X_{n}\right)\right\} & = & \;E_{K_{n,\epsilon }}\left\{\frac{\partial^{2}}{\partial {\tilde{\beta }}^{2}}\rho_{q}\left(Y,F^{-1}\left({\tilde{X}}_{n}^{T}\tilde{\beta }\right)\right)w\left(X_{n}\right)\right\}. \end{array}$$

Therefore,

$$\begin{array}{cc} & \frac{\partial^{2}}{\partial {\tilde{\beta }}^{2}}\;E_{K_{n,\epsilon }}\left\{\rho_{q}\left(Y,F^{-1}\left({\tilde{X}}_{n}^{T}\tilde{\beta }\right)\right)w\left(X_{n}\right)\right\}\\ = & \;E_{K_{n,\epsilon }}\left\{p_{2}\left(Y;{\tilde{X}}_{n}^{T}\tilde{\beta }\right)w\left(X_{n}\right){\tilde{X}}_{n}{\tilde{X}}_{n}^{T}\right\}\\ = & \;E_{K_{n,0}}\left\{p_{2}\left(Y;{\tilde{X}}_{n}^{T}{\tilde{\beta }}_{n,0}\right)w\left(X_{n}\right){\tilde{X}}_{n}{\tilde{X}}_{n}^{T}\right\}\\ & +\;E_{K_{n,0}}\left[\left\{p_{2}\left(Y;{\tilde{X}}_{n}^{T}\tilde{\beta }\right)-p_{2}\left(Y;{\tilde{X}}_{n}^{T}{\tilde{\beta }}_{n,0}\right)\right\}w\left(X_{n}\right){\tilde{X}}_{n}{\tilde{X}}_{n}^{T}\right]\\ & +[\;E_{K_{n,\epsilon }}\left\{p_{2}\left(Y;{\tilde{X}}_{n}^{T}\tilde{\beta }\right)w\left(X_{n}\right){\tilde{X}}_{n}{\tilde{X}}_{n}^{T}\right\}-\;E_{K_{n,0}}\left\{p_{2}\left(Y;{\tilde{X}}_{n}^{T}\tilde{\beta }\right)w\left(X_{n}\right){\tilde{X}}_{n}{\tilde{X}}_{n}^{T}\right\}]\\ = & I_{1}^{*}+I_{2}^{*}+I_{3}^{*}. \end{array}$$

We know that the minimum eigenvalues of $I_{1}^{*}$ are uniformly bounded away from 0,

$$\begin{array}{ccc} ∥I_{2}^{*}∥ & = & ∥\;E_{K_{n,0}}\left\{p_{3}\left(Y;{\tilde{X}}_{n}^{T}{\tilde{\beta }}^{**}\right)w\left(X_{n}\right){\tilde{X}}_{n}{\tilde{X}}_{n}^{T}{\tilde{X}}_{n}^{T}\left(\tilde{\beta }-{\tilde{\beta }}_{n,0}\right)\right\}∥\leq Cp_{n}/n^{1/4}=o\left(1\right)\\ ∥I_{3}^{*}∥ & \leq & \epsilon /\sqrt{n}[∥\;E_{K_{n,0}}\left\{p_{2}\left(Y;{\tilde{X}}_{n}^{T}\tilde{\beta }\right)w\left(X_{n}\right){\tilde{X}}_{n}{\tilde{X}}_{n}^{T}\right\}∥+∥\;E_{J}\left\{p_{2}\left(Y;{\tilde{X}}_{n}^{T}\tilde{\beta }\right)w\left(X_{n}\right){\tilde{X}}_{n}{\tilde{X}}_{n}^{T}\right\}∥]\\ & \leq & Cp_{n}/\sqrt{n}=o\left(1\right). \end{array}$$

Hence, for *n* large enough, $\frac{\partial^{2}}{\partial {\tilde{\beta }}^{2}}\;E_{K_{n,\epsilon }}\left\{\rho_{q}\left(Y,F^{-1}\left({\tilde{X}}_{n}^{T}\tilde{\beta }\right)\right)w\left(X_{n}\right)\right\}$ is positive definite for all $\tilde{\beta }$ such that $∥\tilde{\beta }-{\tilde{\beta }}_{n,0}∥=O\left(n^{-1/4}p_{n}^{-1/2}\right)$. Therefore, there exists a unique minimizer of $\ell_{K_{n,\epsilon }}\left(\tilde{\beta }\right)$ in the $n^{-1/4}p_{n}^{-1/2}$ neighborhood of ${\tilde{\beta }}_{n,0}$ which covers ${\tilde{\beta }}_{n,\epsilon }$. From

$$\begin{array}{ccc} 0 & = & \frac{\partial }{\partial \tilde{\beta }}\;E_{K_{n,\epsilon }}\left\{\rho_{q}\left(Y,F^{-1}\left({\tilde{X}}_{n}^{T}\tilde{\beta }\right)\right)w\left(X_{n}\right)\right\}{|}_{\tilde{\beta }={\tilde{\beta }}_{n,\epsilon }}=\;E_{K_{n,\epsilon }}\left\{\frac{\partial }{\partial \tilde{\beta }}\rho_{q}\left(Y,F^{-1}\left({\tilde{X}}_{n}^{T}\tilde{\beta }\right)\right){|}_{\tilde{\beta }={\tilde{\beta }}_{n,\epsilon }}w\left(X_{n}\right)\right\}\\ & = & \;E_{K_{n,\epsilon }}\left\{p_{1}\left(Y;{\tilde{X}}_{n}^{T}{\tilde{\beta }}_{n,\epsilon }\right)w\left(X_{n}\right){\tilde{X}}_{n}\right\}, \end{array}$$

we know $T\left(K_{n,\epsilon }\right)={\tilde{\beta }}_{n,\epsilon }$. From the definition of T(·), it’s easy to see that $T(K_{n,\epsilon })$ is unique. ☐

**Lemma A2** ($∥T\left(K_{n}\right)-T(K_{n,\epsilon })∥$)**.** *Assume Conditions* A0–A7 *and* B4. *For $K_{n,\epsilon }$ in Equation* (10), $\ell_{K}\left(\cdot \right)$ *in Equation* (11) *and* T(·) *in Equation* (12), *if $p_{n}^{4}/n\to 0$ as n→∞ and the distribution of $\left(X_{n},Y\right)$ is $K_{n,\epsilon }$, then there exists a unique local minimizer ${\hat{\tilde{\beta }}}_{n}$ of $\ell_{K_{n}}\left(\tilde{\beta }\right)$ such that $∥{\hat{\tilde{\beta }}}_{n}-T(K_{n,\epsilon })∥=O_{P}\left(\sqrt{p_{n}/n}\right)$. Furthermore, $∥{\hat{\tilde{\beta }}}_{n}-{\tilde{\beta }}_{n,0}∥=O_{P}\left(\sqrt{p_{n}/n}\right)$ and $T\left(K_{n}\right)=\hat{\tilde{\beta }}$.*

**Proof.** Let $r_{n}=\sqrt{p_{n}/n}$ and ${\tilde{u}}_{n}={\left(u_{0},u_{1},\ldots ,u_{p_{n}}\right)}^{T}\in R^{p_{n}+1}$. To show the existence of the estimator, it suffices to show that for any given κ>0, there exists a sufficiently large constant $C_{\kappa }$ such that, for large *n* we have

(A4) $$\begin{array}{c} P\left\{\underset{∥{\tilde{u}}_{n}∥=C_{\kappa }}{\inf}\ell_{K_{n}}\left(T(K_{n,\epsilon })+r_{n}{\tilde{u}}_{n}\right)>\ell_{K_{n}}\left(T(K_{n,\epsilon })\right)\right\}\geq 1-\kappa . \end{array}$$

This implies that with probability at least 1−κ, there exists a local minimizer ${\hat{\tilde{\beta }}}_{n}$ of $\ell_{K_{n}}\left(\tilde{\beta }\right)$ in the ball $\left\{T(K_{n,\epsilon })+r_{n}{\tilde{u}}_{n}:∥{\tilde{u}}_{n}∥\leq C_{\kappa }\right\}$. To show Equation (A4), consider

$$\begin{array}{ccc} \ell_{K_{n}}\left(T(K_{n,\epsilon })+r_{n}{\tilde{u}}_{n}\right)-\ell_{K_{n}}\left(T(K_{n,\epsilon })\right) & = & \frac{1}{n}\sum_{i=1}^{n}\{\rho_{q}\left(Y_{i},F^{-1}\left({\tilde{X}}_{ni}^{T}\left(T(K_{n,\epsilon })+r_{n}{\tilde{u}}_{n}\right)\right)\right)w\left(X_{ni}\right)\\ & & -\rho_{q}\left(Y_{i},F^{-1}\left({\tilde{X}}_{ni}^{T}T(K_{n,\epsilon })\right)\right)w\left(X_{ni}\right)\}\\ & \equiv & I_{1}, \end{array}$$

where $∥{\tilde{u}}_{n}∥=C_{\kappa }$. By Taylor expansion,

(A5) $$\begin{array}{c} I_{1}=I_{1,1}+I_{1,2}+I_{1,3}, \end{array}$$

where

$$\begin{array}{ccc} I_{1,1} & = & r_{n}/n\sum_{i=1}^{n}p_{1}\left(Y_{i};{\tilde{X}}_{ni}^{T}T(K_{n,\epsilon })\right)w\left(X_{ni}\right){\tilde{X}}_{ni}^{T}{\tilde{u}}_{n},\\ I_{1,2} & = & r_{n}^{2}/\left(2n\right)\sum_{i=1}^{n}p_{2}\left(Y_{i};{\tilde{X}}_{ni}^{T}T(K_{n,\epsilon })\right)w\left(X_{ni}\right){\left({\tilde{X}}_{ni}^{T}{\tilde{u}}_{n}\right)}^{2},\\ I_{1,3} & = & r_{n}^{3}/\left(6n\right)\sum_{i=1}^{n}p_{3}\left(Y_{i};{\tilde{X}}_{ni}^{T}{\tilde{\beta }}_{n}^{*}\right)w\left(X_{ni}\right){\left({\tilde{X}}_{ni}^{T}{\tilde{u}}_{n}\right)}^{3} \end{array}$$

for ${\tilde{\beta }}_{n}^{*}$ located between $T(K_{n,\epsilon })$ and $T(K_{n,\epsilon })+r_{n}{\tilde{u}}_{n}$. Since $∥T(K_{n,\epsilon })-{\tilde{\beta }}_{n,0}∥=O\left(\sqrt{p_{n}/n}\right)=o\left(1\right)$, the large open set considered in Condition A6 contains $T(K_{n,\epsilon })$ when *n* is large enough, say n≥N where *N* is a positive constant. Therefore, for any fixed n≥N, there exists a bounded open subset of $R^{p_{n}+1}$ containing $T(K_{n,\epsilon })$ such that for all $\tilde{\beta }$ in this set, $∥p_{1}\left(Y;{\tilde{X}}_{n}^{T}\tilde{\beta }\right)w\left(X_{n}\right){\tilde{X}}_{n}∥\leq C∥{\tilde{X}}_{n}∥$ which is integrable with respect to $K_{n,\epsilon }$, where *C* is a positive constant. Thus, for n≥N,

(A6) $$\begin{array}{c} 0=\frac{\partial }{\partial \tilde{\beta }}\;E_{K_{n,\epsilon }}\left\{\rho_{q}\left(Y,F^{-1}\left({\tilde{X}}_{n}^{T}\tilde{\beta }\right)\right)w\left(X_{n}\right)\right\}{|}_{\tilde{\beta }=T(K_{n,\epsilon })}=\;E_{K_{n,\epsilon }}\left\{p_{1}\left(Y;{\tilde{X}}_{n}^{T}T(K_{n,\epsilon })\right)w\left(X_{n}\right){\tilde{X}}_{n}\right\}. \end{array}$$

Hence,

$$\begin{array}{c} |I_{1,1}|\leq r_{n}∥\frac{1}{n}\sum_{i=1}^{n}p_{1}\left(Y_{i};{\tilde{X}}_{ni}^{T}T(K_{n,\epsilon })\right)w\left(X_{ni}\right){\tilde{X}}_{ni}∥∥{\tilde{u}}_{n}∥=O_{P}\left(r_{n}\sqrt{p_{n}/n}\right)∥{\tilde{u}}_{n}∥. \end{array}$$

For $I_{1,2}$ in Equation (A5),

$$\begin{array}{ccc} I_{1,2} & = & \frac{r_{n}^{2}}{2n}\sum_{i=1}^{n}\;E_{K_{n,\epsilon }}\left\{p_{2}\left(Y_{i};{\tilde{X}}_{ni}^{T}T(K_{n,\epsilon })\right)w\left(X_{ni}\right){\left({\tilde{X}}_{ni}^{T}{\tilde{u}}_{n}\right)}^{2}\right\}\\ & & +\frac{r_{n}^{2}}{2n}\sum_{i=1}^{n}[p_{2}\left(Y_{i};{\tilde{X}}_{ni}^{T}T(K_{n,\epsilon })\right)w\left(X_{ni}\right){\left({\tilde{X}}_{ni}^{T}{\tilde{u}}_{n}\right)}^{2}\\ & & -\;E_{K_{n,\epsilon }}\left\{p_{2}\left(Y_{i};{\tilde{X}}_{ni}^{T}T(K_{n,\epsilon })\right)w\left(X_{ni}\right){\left({\tilde{X}}_{ni}^{T}{\tilde{u}}_{n}\right)}^{2}\right\}]\\ & \equiv & I_{1,2,1}+I_{1,2,2}, \end{array}$$

where $I_{1,2,1}=2^{-1}r_{n}^{2}{\tilde{u}}_{n}^{T}H_{n,\epsilon }{\tilde{u}}_{n}$. Meanwhile, we have

$$\begin{array}{ccc} |I_{1,2,2}| & \leq & \frac{r_{n}^{2}}{2}∥\frac{1}{n}\sum_{i=1}^{n}[p_{2}\left(Y_{i};{\tilde{X}}_{ni}^{T}T(K_{n,\epsilon })\right)w\left(X_{ni}\right){\tilde{X}}_{ni}{\tilde{X}}_{ni}^{T}\\ & & -\;E_{K_{n,\epsilon }}\left\{p_{2}\left(Y_{i};{\tilde{X}}_{ni}^{T}T(K_{n,\epsilon })\right)w\left(X_{ni}\right){\tilde{X}}_{ni}{\tilde{X}}_{ni}^{T}\right\}]{∥}_{F}{∥{\tilde{u}}_{n}∥}^{2}\\ & = & r_{n}^{2}O_{P}\left(p_{n}/\sqrt{n}\right){∥{\tilde{u}}_{n}∥}^{2}. \end{array}$$

Thus,

(A7) $$\begin{array}{c} I_{1,2}=2^{-1}r_{n}^{2}{\tilde{u}}_{n}^{T}H_{n,\epsilon }{\tilde{u}}_{n}+O_{P}\left(r_{n}^{2}p_{n}/\sqrt{n}\right){∥{\tilde{u}}_{n}∥}^{2}. \end{array}$$

For $I_{1,3}$ in Equation (A5), we observe that

$$\begin{array}{c} |I_{1,3}|\leq Cr_{n}^{3}\frac{1}{n}\sum_{i=1}^{n}|p_{3}\left(Y_{i};{\tilde{X}}_{ni}^{T}{\tilde{\beta }}_{n}^{*}\right)|w\left(X_{ni}\right)|{\tilde{X}}_{ni}^{T}{\tilde{u}}_{n}{|}^{3}=O_{P}\left(r_{n}^{3}p_{n}^{3/2}\right){∥{\tilde{u}}_{n}∥}^{3}. \end{array}$$

We will show that the minimum eigenvalue of $H_{n,\epsilon }$ is uniformly bounded away from 0. $H_{n,\epsilon }=\left(1-\epsilon /\sqrt{n}\right)H_{n,K_{n,0},T\left(K_{n,\epsilon }\right)}+\epsilon /\sqrt{n}H_{n,J,T(K_{n,\epsilon })}$. Note

$$\begin{array}{cc} & ∥H_{n,K_{n,0},T\left(K_{n,\epsilon }\right)}-H_{n}∥\\ = & ∥\;E_{K_{n,0}}\left[\left\{p_{2}\left(Y;{\tilde{X}}_{n}^{T}T\left(K_{n,\epsilon }\right)\right)-p_{2}\left(Y;{\tilde{X}}_{n}^{T}{\tilde{\beta }}_{n,0}\right)\right\}w\left(X_{n}\right){\tilde{X}}_{n}{\tilde{X}}_{n}^{T}\right]∥\\ = & ∥\;E_{K_{n,0}}\left[p_{3}\left(Y;{\tilde{X}}_{n}^{T}{\tilde{\beta }}_{n}^{**}\right)w\left(X_{n}\right){\tilde{X}}_{n}{\tilde{X}}_{n}^{T}{\tilde{X}}_{n}^{T}\left\{T\left(K_{n,\epsilon }\right)-{\tilde{\beta }}_{n,0}\right\}\right]∥=O\left(p_{n}^{2}/\sqrt{n}\right). \end{array}$$

Since the eigenvalues of $H_{n}$ are uniformly bounded away from 0, so are those of $H_{n,K_{n,0},T\left(K_{n,\epsilon }\right)}$ and $H_{n,\epsilon }$. We can choose some large $C_{\kappa }$ such that $I_{1,1}$ and $I_{1,3}$ are both dominated by the first term of $I_{1,2}$ in Equation (A7), which is positive by the eigenvalue assumption. This implies Equation (A4). Next we show the uniqueness of $\hat{\tilde{\beta }}$. For all $\tilde{\beta }$ such that $∥\tilde{\beta }-T(K_{n,\epsilon })∥=O\left(n^{-1/4}p_{n}^{-1/2}\right)$,

$$\begin{array}{cc} & \frac{1}{n}\sum_{i=1}^{n}p_{2}\left(Y_{i};{\tilde{X}}_{ni}^{T}\tilde{\beta }\right)w\left(X_{ni}\right){\tilde{X}}_{ni}{\tilde{X}}_{ni}^{T}\\ = & \;E_{K_{n,0}}\left\{p_{2}\left(Y;{\tilde{X}}_{n}^{T}{\tilde{\beta }}_{n,0}\right)w\left(X_{n}\right){\tilde{X}}_{n}X_{n}^{T}\right\}\\ & +\;E_{K_{n,0}}\left[\left\{p_{2}\left(Y;{\tilde{X}}_{n}^{T}\tilde{\beta }\right)-p_{2}\left(Y;{\tilde{X}}_{n}^{T}{\tilde{\beta }}_{n,0}\right)\right\}w\left(X_{n}\right){\tilde{X}}_{n}X_{n}^{T}\right]\\ & +[\;E_{K_{n,\epsilon }}\left\{p_{2}\left(Y;{\tilde{X}}_{n}^{T}\tilde{\beta }\right)w\left(X_{n}\right){\tilde{X}}_{n}X_{n}^{T}\right\}-\;E_{K_{n,0}}\left\{p_{2}\left(Y;{\tilde{X}}_{n}^{T}\tilde{\beta }\right)w\left(X_{n}\right){\tilde{X}}_{n}X_{n}^{T}\right\}]\\ & +\left[\frac{1}{n}\sum_{i=1}^{n}p_{2}\left(Y_{i};{\tilde{X}}_{ni}^{T}\tilde{\beta }\right))w\left(X_{ni}\right){\tilde{X}}_{ni}{\tilde{X}}_{ni}^{T}-\;E_{K_{n,\epsilon }}\left\{p_{2}\left(Y;{\tilde{X}}_{n}^{T}\tilde{\beta }\right)w\left(X_{n}\right){\tilde{X}}_{n}X_{n}^{T}\right\}\right]\\ = & I_{1}^{*}+I_{2}^{*}+I_{3}^{*}+I_{4}^{*}. \end{array}$$

We know that the minimum eigenvalues of $I_{1}^{*}$ are uniformly bounded away from 0. Following the proof of Lemma A1, we have $∥I_{2}^{*}∥=o\left(1\right)$ and $∥I_{3}^{*}∥=o\left(1\right)$. It’s easy to see $∥I_{4}^{*}∥=O_{P}\left(p_{n}/\sqrt{n}\right)$. Hence, for *n* large enough, $\frac{\partial^{2}}{\partial {\tilde{\beta }}^{2}}\ell_{K_{n}}\left(\tilde{\beta }\right)$ is positive definite with high probability for all $\tilde{\beta }$ such that $∥\tilde{\beta }-{\tilde{\beta }}_{n,0}∥=O\left(n^{-1/4}p_{n}^{-1/2}\right)$. Therefore, there exists a unique minimizer of $\ell_{K_{n}}\left(\tilde{\beta }\right)$ in the $n^{-1/4}p_{n}^{-1/2}$ neighborhood of $T(K_{n,\epsilon })$ which covers $\hat{\tilde{\beta }}$. ☐

**Lemma A3** ($∥A_{n}\left\{T\left(K_{n,\epsilon }\right)-{\tilde{\beta }}_{n,0}\right\}∥$)**.** *Assume Conditions* A0–A7 *and* B4. *For $K_{n,\epsilon }$ in Equation* (10) *and T(·) in Equation* (12), *if $p_{n}^{5}/n\to 0$ as n→∞, the distribution of $\left(X_{n},Y\right)$ is $K_{n,\epsilon }$ and $\;E_{J}(∥w\left(X_{n}\right)X_{n}∥)\leq C$, then*

$$\begin{array}{c} \sqrt{n}A_{n}\left\{T(K_{n,\epsilon })-{\tilde{\beta }}_{n,0}\right\}=O\left(1\right), \end{array}$$

*where $A_{n}$ is any given $k\times (p_{n}+1)$ matrix such that $A_{n}A_{n}^{T}\to G$, with G being a k×k positive-definite matrix and k is a fixed integer.*

**Proof.** Taylor’s expansion yields

$$\begin{array}{ccc} 0 & = & \;E_{K_{n,\epsilon }}\left\{p_{1}\left(Y;{\tilde{X}}_{n}^{T}T\left(K_{n,\epsilon }\right)\right)w\left(X_{n}\right){\tilde{X}}_{n}\right\}\\ & = & \;E_{K_{n,\epsilon }}\left\{p_{1}\left(Y;{\tilde{X}}_{n}^{T}{\tilde{\beta }}_{n,0}\right)w\left(X_{n}\right){\tilde{X}}_{n}\right\}\\ & & +\;E_{K_{n,\epsilon }}\left\{p_{2}\left(Y;{\tilde{X}}_{n}^{T}{\tilde{\beta }}_{n,0}\right)w\left(X_{n}\right){\tilde{X}}_{n}{\tilde{X}}_{n}^{T}\right\}\left\{T\left(K_{n,\epsilon }\right)-{\tilde{\beta }}_{n,0}\right\}\\ & & +1/2\;E_{K_{n,\epsilon }}\left(p_{3}\left(Y;{\tilde{X}}_{n}^{T}{\tilde{\beta }}_{n}^{*}\right)w\left(X_{n}\right){\tilde{X}}_{n}{\left[{\tilde{X}}_{n}^{T}\left\{T\left(K_{n,\epsilon }\right)-{\tilde{\beta }}_{n,0}\right\}\right]}^{2}\right)\\ & = & I_{1}+I_{2}\left\{T\left(K_{n,\epsilon }\right)-{\tilde{\beta }}_{n,0}\right\}+I_{3}, \end{array}$$

where ${\tilde{\beta }}_{n}^{*}$ lies between $T(K_{n,\epsilon })$ and ${\tilde{\beta }}_{n,0}$. Below, we will show

$$\begin{array}{c} ∥I_{1}∥=O\left(1/\sqrt{n}\right),\;∥I_{2}-H_{n}∥=O\left(p_{n}/\sqrt{n}\right),\;∥I_{3}∥=O\left(p_{n}^{5/2}/n\right). \end{array}$$

First, $∥I_{1}∥=\epsilon /\sqrt{n}∥\;E_{J}\left\{p_{1}\left(Y;{\tilde{X}}_{n}^{T}T\left(K_{n,\epsilon }\right)\right)w\left(X_{n}\right){\tilde{X}}_{n}\right\}∥\leq C\epsilon /\sqrt{n}\;E_{J}(∥w\left(X_{n}\right)X_{n}∥)=O\left(1/\sqrt{n}\right)$. Following the proof of $I_{3}^{*}$ in Lemma A1, $∥I_{2}-H_{n}∥=O\left(p_{n}/\sqrt{n}\right)$. Since $∥T\left(K_{n,\epsilon }\right)-{\tilde{\beta }}_{n,0}∥=O\left(\sqrt{p_{n}/n}\right)$, we have $∥I_{3}∥=O\left(p_{n}^{5/2}/n\right)$. Therefore, $\sqrt{n}A_{n}\left\{T\left(K_{n,\epsilon }\right)-{\tilde{\beta }}_{n,0}\right\}=-\sqrt{n}A_{n}H_{n}^{-1}I_{1}+o\left(1\right),$ which completes the proof. ☐

**Lemma A4** (asymptotic normality of $T\left(K_{n}\right)-T\left(K_{n,\epsilon }\right)$)**.** *Assume Conditions* A0–A8 *and* B4. *If $p_{n}^{5}/n\to 0$ as n→∞ and the distribution of $\left(X_{n},Y\right)$ is $K_{n,\epsilon }$, then*

$$\begin{array}{c} \sqrt{n}{\left\{U\left(K_{n,\epsilon }\right)\right\}}^{-1/2}A_{n}\left\{T\left(K_{n}\right)-T\left(K_{n,\epsilon }\right)\right\}\overset{L}{⟶}N\left(0,I_{k}\right), \end{array}$$

*where $U\left(K_{n,\epsilon }\right)=A_{n}H_{n,\epsilon }^{-1}\Omega_{n,\epsilon }H_{n,\epsilon }^{-1}A_{n}^{T}$, $A_{n}$ is any given $k\times (p_{n}+1)$ matrix such that $A_{n}A_{n}^{T}\to G$, with G being a k×k positive-definite matrix, k is a fixed integer.*

**Proof.** We will first show that

(A8) $$\begin{array}{c} T\left(K_{n}\right)-T\left(K_{n,\epsilon }\right)=-\frac{1}{n}H_{n,\epsilon }^{-1}\sum_{i=1}^{n}p_{1}\left(Y_{i};{\tilde{X}}_{ni}^{T}T\left(K_{n,\epsilon }\right)\right)w\left(X_{ni}\right){\tilde{X}}_{ni}+o_{P}\left(n^{-1/2}\right). \end{array}$$

From $\frac{\partial \ell_{K_{n}}\left(\tilde{\beta }\right)}{\partial \tilde{\beta }}{|}_{\tilde{\beta }=T\left(K_{n}\right)}=0$, Taylor’s expansion yields

(A9) $$\begin{array}{ccc} 0 & = & \left\{\frac{1}{n}\sum_{i=1}^{n}p_{1}\left(Y_{i};{\tilde{X}}_{ni}^{T}T\left(K_{n,\epsilon }\right)\right)w\left(X_{ni}\right){\tilde{X}}_{ni}\right\}\\ & & +\left\{\frac{1}{n}\sum_{i=1}^{n}p_{2}\left(Y_{i};{\tilde{X}}_{ni}^{T}T\left(K_{n,\epsilon }\right)\right)w\left(X_{ni}\right){\tilde{X}}_{ni}{\tilde{X}}_{ni}^{T}\right\}\left\{T\left(K_{n}\right)-T\left(K_{n,\epsilon }\right)\right\}\\ & & +\frac{1}{2n}\sum_{i=1}^{n}p_{3}\left(Y_{i};{\tilde{X}}_{ni}^{T}{\tilde{\beta }}_{n}^{*}\right)w\left(X_{ni}\right){\left[{\tilde{X}}_{ni}^{T}\left\{T\left(K_{n}\right)-T\left(K_{n,\epsilon }\right)\right\}\right]}^{2}{\tilde{X}}_{ni}\\ & \equiv & \left\{\frac{1}{n}\sum_{i=1}^{n}p_{1}\left(Y_{i};{\tilde{X}}_{ni}^{T}T\left(K_{n,\epsilon }\right)\right)w\left(X_{ni}\right){\tilde{X}}_{ni}\right\}+I_{2}\left\{T\left(K_{n}\right)-T\left(K_{n,\epsilon }\right)\right\}+I_{3}, \end{array}$$

where ${\tilde{\beta }}_{n}^{*}$ lies between $T(K_{n,\epsilon })$ and $T(K_{n})$. Below, we will show

$$\begin{array}{c} ∥I_{2}-H_{n,\epsilon }∥=O_{P}\left(p_{n}/\sqrt{n}\right),\;∥I_{3}∥=O_{P}\left(p_{n}^{5/2}/n\right). \end{array}$$

Similar arguments for the proof of $I_{1,2}$ of Lemma A2, we have $∥I_{2}-H_{n,\epsilon }∥=O_{P}\left(p_{n}/\sqrt{n}\right)$. Second, a similar proof used for $I_{1,3}^{*}$ in Equation (A5) gives $∥I_{3}∥=O_{P}\left(p_{n}^{5/2}/n\right).$ Third, by Equation (A9) and $∥T\left(K_{n}\right)-T\left(K_{n,\epsilon }\right)∥=O_{P}\left(\sqrt{p_{n}/n}\right)$, we see that

$$\begin{array}{c} H_{n,\epsilon }\left\{T\left(K_{n}\right)-T\left(K_{n,\epsilon }\right)\right\}=-\frac{1}{n}\sum_{i=1}^{n}p_{1}\left(Y_{i};{\tilde{X}}_{ni}^{T}T\left(K_{n,\epsilon }\right)\right)w\left(X_{ni}\right){\tilde{X}}_{ni}+u_{n}, \end{array}$$

where $∥u_{n}∥=O_{P}\left(p_{n}^{5/2}/n\right)=o_{P}\left(n^{-1/2}\right)$. From the proof of Lemma A2, the eigenvalues of $H_{n,\epsilon }$ are uniformly bounded away from 0 and we complete the proof of Equation (A8). Following the proof for the bounded eigenvalues of $H_{n,\epsilon }$ in Lemma A2, we can show that the eigenvalues of $\Omega_{n,\epsilon }$ are uniformly bounded away from 0. Hence, the eigenvalues of $H_{n,\epsilon }^{-1}\Omega_{n,\epsilon }H_{n,\epsilon }^{-1}$ are uniformly bounded away from 0, as are the eigenvalues of $U(K_{n,\epsilon })$. From Equation (A8), we see that

$$\begin{array}{c} A_{n}\left\{T\left(K_{n}\right)-T\left(K_{n,\epsilon }\right)\right\}=-\frac{1}{n}A_{n}H_{n,\epsilon }^{-1}\sum_{i=1}^{n}p_{1}\left(Y_{i};{\tilde{X}}_{ni}^{T}T\left(K_{n,\epsilon }\right)\right)w\left(X_{ni}\right){\tilde{X}}_{ni}+o_{P}\left(n^{-1/2}\right). \end{array}$$

It follows that

$$\begin{array}{c} \sqrt{n}{\left\{U\left(K_{n,\epsilon }\right)\right\}}^{-1/2}A_{n}\left\{T\left(K_{n}\right)-T\left(K_{n,\epsilon }\right)\right\}=\sum_{i=1}^{n}R_{ni}+o_{P}\left(1\right), \end{array}$$

where $R_{ni}=-n^{-1/2}{\left\{U\left(K_{n,\epsilon }\right)\right\}}^{-1/2}A_{n}H_{n,\epsilon }^{-1}p_{1}\left(Y_{i};{\tilde{X}}_{ni}^{T}T\left(K_{n,\epsilon }\right)\right)w\left(X_{ni}\right){\tilde{X}}_{ni}$. Following (A6) in Lemma A2, one can show that $\;E_{K_{n,\epsilon }}\left(R_{ni}\right)=0$ for *n* large enough. To show $\sum_{i=1}^{n}R_{ni}\overset{L}{⟶}N\left(0,I_{k}\right)$, we apply the Lindeberg-Feller central limit theorem in [26]. Specifically, we check (I) $\sum_{i=1}^{n}{\mathrm{cov}}_{K_{n,\epsilon }}\left(R_{ni}\right)\to I_{k}$; (II) $\sum_{i=1}^{n}\;E_{K_{n,\epsilon }}(∥R_{ni}{∥}^{2+\delta })=o\left(1\right)$ for some δ>0. Condition (I) is straightforward since $\sum_{i=1}^{n}{\mathrm{cov}}_{K_{n,\epsilon }}\left(R_{ni}\right)={\left\{U\left(K_{n,\epsilon }\right)\right\}}^{-1/2}U\left(K_{n,\epsilon }\right){\left\{U\left(K_{n,\epsilon }\right)\right\}}^{-1/2}=I_{k}$. To check condition (II), we can show that $\;E_{K_{n,\epsilon }}(∥R_{ni}{∥}^{2+\delta })=O\left({\left(p_{n}/n\right)}^{(2+\delta )/2}\right)$. This yields $\sum_{i=1}^{n}\;E_{K_{n,\epsilon }}(∥R_{ni}{∥}^{2+\delta })\leq O\left(p_{n}^{(2+\delta )/2}/n^{\delta /2}\right)=o\left(1\right)$. Hence

$$\begin{array}{c} \sqrt{n}{\left\{U\left(K_{n,\epsilon }\right)\right\}}^{-1/2}A_{n}\left\{T\left(K_{n}\right)-T\left(K_{n,\epsilon }\right)\right\}\overset{L}{⟶}N\left(0,I_{k}\right). \end{array}$$

Thus, we complete the proof. ☐

**Lemma A5** (asymptotic covariance matrices $U(K_{n,\epsilon })$ and $U_{n}$)**.** *Assume Conditions* A0–A9 *and* B4. *If $p_{n}^{4}/n\to 0$ as n→∞, then*

$$\begin{array}{c} ∥U_{n}^{-1/2}{\left\{U\left(K_{n,\epsilon }\right)\right\}}^{1/2}-I_{k}∥=O\left(p_{n}/n^{1/4}\right), \end{array}$$

*where $U\left(K_{n,\epsilon }\right)=A_{n}H_{n,\epsilon }^{-1}\Omega_{n,\epsilon }H_{n,\epsilon }^{-1}A_{n}^{T}$, $A_{n}$ is any given $k\times (p_{n}+1)$ matrix such that $A_{n}A_{n}^{T}\to G$, with G being a k×k positive-definite matrix, and k is a fixed integer.*

**Proof.** Note that

$$\begin{array}{cc} & ∥{\left\{U\left(K_{n,\epsilon }\right)\right\}}^{1/2}-U_{n}^{1/2}{∥}^{2}\leq ∥U\left(K_{n,\epsilon }\right)-U_{n}∥\\ \leq & ∥H_{n,\epsilon }^{-1}\Omega_{n,\epsilon }H_{n,\epsilon }^{-1}-H_{n}^{-1}\Omega_{n}H_{n}^{-1}∥∥A_{n}{∥}_{F}^{2}. \end{array}$$

Since $∥A_{n}{∥}_{F}^{2}\to \mathrm{tr}\left(G\right)$, it suffices to prove that $∥H_{n,\epsilon }^{-1}\Omega_{n,\epsilon }H_{n,\epsilon }^{-1}-H_{n}^{-1}\Omega_{n}H_{n}^{-1}∥=O\left(p_{n}^{2}/\sqrt{n}\right)$. First, we prove $∥H_{n,\epsilon }-H_{n}∥=O\left(p_{n}^{2}/\sqrt{n}\right)$. Note that

$$\begin{array}{ccc} H_{n,\epsilon }-H_{n} & = & \;E_{K_{n,\epsilon }}\left[\left\{p_{2}\left(Y;{\tilde{X}}_{n}^{T}T\left(K_{n,\epsilon }\right)\right)-p_{2}\left(Y;{\tilde{X}}_{n}^{T}{\tilde{\beta }}_{n,0}\right)\right\}w\left(X_{n}\right){\tilde{X}}_{n}{\tilde{X}}_{n}^{T}\right]\\ & & +[\;E_{K_{n,\epsilon }}\left\{p_{2}\left(Y;{\tilde{X}}_{n}^{T}{\tilde{\beta }}_{n,0}\right)w\left(X_{n}\right){\tilde{X}}_{n}{\tilde{X}}_{n}^{T}\right\}-H_{n}]\\ & = & \;E_{K_{n,\epsilon }}\left[p_{3}\left(Y;{\tilde{X}}_{n}^{T}{\tilde{\beta }}^{*}\right)w\left(X_{n}\right){\tilde{X}}_{n}{\tilde{X}}_{n}^{T}{\tilde{X}}_{n}^{T}\left\{T\left(K_{n,\epsilon }\right)-{\tilde{\beta }}_{n,0}\right\}\right]\\ & & +[\;E_{K_{n,\epsilon }}\left\{p_{2}\left(Y;{\tilde{X}}_{n}^{T}{\tilde{\beta }}_{n,0}\right)w\left(X_{n}\right){\tilde{X}}_{n}{\tilde{X}}_{n}^{T}\right\}-H_{n}]\\ & \equiv & I_{1}+I_{2}. \end{array}$$

We know that $∥I_{1}∥=O\left(p_{n}^{2}/\sqrt{n}\right)$ and $∥I_{2}∥=O\left(p_{n}/\sqrt{n}\right)$. Thus, $∥I_{1}∥=O\left(p_{n}^{2}/\sqrt{n}\right)$. Second, we show $∥\Omega_{n,\epsilon }-\Omega_{n}∥=O\left(p_{n}^{2}/\sqrt{n}\right)$. It is easy to see that

$$\begin{array}{ccc} \Omega_{n,\epsilon }-\Omega_{n} & = & \;E_{K_{n,\epsilon }}\left[\left\{p_{1}^{2}\left(Y;{\tilde{X}}_{n}^{T}T\left(K_{n,\epsilon }\right)\right)-p_{1}^{2}\left(Y;{\tilde{X}}_{n}^{T}{\tilde{\beta }}_{n,0}\right)\right\}w^{2}\left(X_{n}\right){\tilde{X}}_{n}{\tilde{X}}_{n}^{T}\right]\\ & & +[\;E_{K_{n,\epsilon }}\left\{p_{1}^{2}\left(Y;{\tilde{X}}_{n}^{T}{\tilde{\beta }}_{n,0}\right)w^{2}\left(X_{n}\right){\tilde{X}}_{n}{\tilde{X}}_{n}^{T}\right\}-\Omega_{n}]\\ & = & \Delta_{1,1}+\Delta_{1,2}, \end{array}$$

where $∥\Delta_{1,1}∥=O\left(p_{n}^{2}/\sqrt{n}\right)$ and $∥\Delta_{1,2}∥=O\left(p_{n}/\sqrt{n}\right)$. We observe that $∥\Omega_{n,\epsilon }-\Omega_{n}∥=O\left(p_{n}^{2}/\sqrt{n}\right)$. Third, we show $∥H_{n,\epsilon }^{-1}\Omega_{n,\epsilon }H_{n,\epsilon }^{-1}-H_{n}^{-1}\Omega_{n}H_{n}^{-1}∥=O\left(p_{n}^{2}/\sqrt{n}\right)$. Note $H_{n,\epsilon }^{-1}\Omega_{n,\epsilon }H_{n,\epsilon }^{-1}-H_{n}^{-1}\Omega_{n}H_{n}^{-1}=L_{1}+L_{2}+L_{3}$, where $L_{1}=H_{n,\epsilon }^{-1}\left(\Omega_{n,\epsilon }-\Omega_{n}\right)H_{n,\epsilon }^{-1}$, $L_{2}=H_{n,\epsilon }^{-1}\left(H_{n}-H_{n,\epsilon }\right)H_{n}^{-1}\Omega_{n}H_{n,\epsilon }^{-1}$ and $L_{3}=H_{n}^{-1}\Omega_{n}H_{n,\epsilon }^{-1}\left(H_{n}-H_{n,\epsilon }\right)H_{n}^{-1}$. Under Conditions A7 and A9, it is straightforward to see that $∥H_{n,\epsilon }^{-1}∥=O\left(1\right)$, $∥H_{n}^{-1}∥=O\left(1\right)$ and $∥H_{n}^{-1}\Omega_{n}∥=O\left(1\right)$. Since $∥L_{1}∥\leq ∥H_{n,\epsilon }^{-1}∥∥\Omega_{n,\epsilon }-\Omega_{n}∥∥H_{n,\epsilon }^{-1}∥$, we conclude $∥L_{1}∥=O\left(p_{n}^{2}/\sqrt{n}\right)$, and similarly $∥L_{2}∥=O\left(p_{n}^{2}/\sqrt{n}\right)$ and $∥L_{3}∥=O\left(p_{n}^{2}/\sqrt{n}\right)$. Hence, $∥H_{n,\epsilon }^{-1}\Omega_{n,\epsilon }H_{n,\epsilon }^{-1}-H_{n}^{-1}\Omega_{n}H_{n}^{-1}∥=O\left(p_{n}^{2}/\sqrt{n}\right)$. Thus, we can conclude that $∥U\left(K_{n,\epsilon }\right)-U_{n}∥=O\left(p_{n}^{2}/\sqrt{n}\right)$ and that the eigenvalues of $U(K_{n,\epsilon })$ and $U_{n}$ are uniformly bounded away from 0 and *∞*. Consequently, $∥{\left\{U\left(K_{n,\epsilon }\right)\right\}}^{1/2}-U_{n}^{1/2}∥=O\left(p_{n}/n^{1/4}\right)$ and proof is finished. ☐

**Lemma A6** (asymptotic covariance matrices $U(K_{n})$ and $U(K_{n,\epsilon })$)**.** *Assume Conditions* A0–A9 *and* B4. *If $p_{n}^{4}/n\to 0$n→∞ and the distribution of $\left(X_{n},Y\right)$ is $K_{n,\epsilon }$, then*

$$\begin{array}{c} ∥{\left\{U\left(K_{n}\right)\right\}}^{-1/2}{\left\{U\left(K_{n,\epsilon }\right)\right\}}^{1/2}-I_{k}∥=O_{P}\left(p_{n}/n^{1/4}\right), \end{array}$$

*where $U\left(K_{n,\epsilon }\right)=A_{n}H_{n,\epsilon }^{-1}\Omega_{n,\epsilon }H_{n,\epsilon }^{-1}A_{n}^{T},$$U\left(K_{n}\right)=A_{n}{\hat{H}}_{n}^{-1}{\hat{\Omega }}_{n}{\hat{H}}_{n}^{-1}A_{n}^{T},$$A_{n}$ is any given $k\times (p_{n}+1)$ matrix such that $A_{n}A_{n}^{T}\to G$, with G being a k×k positive-definite matrix, and k is a fixed integer.*

**Proof.** Note that $∥{\left\{U\left(K_{n}\right)\right\}}^{1/2}-{\left\{U\left(K_{n,\epsilon }\right)\right\}}^{1/2}{∥}^{2}\leq ∥U\left(K_{n}\right)-U\left(K_{n,\epsilon }\right)∥\leq ∥{\hat{H}}_{n}^{-1}{\hat{\Omega }}_{n}{\hat{H}}_{n}^{-1}-H_{n,\epsilon }^{-1}\Omega_{n,\epsilon }H_{n,\epsilon }^{-1}∥∥A_{n}{∥}_{F}^{2}$. Since $∥A_{n}{∥}_{F}^{2}\to \mathrm{tr}\left(G\right)$, it suffices to prove that $∥{\hat{H}}_{n}^{-1}{\hat{\Omega }}_{n}{\hat{H}}_{n}^{-1}-H_{n,\epsilon }^{-1}\Omega_{n,\epsilon }H_{n,\epsilon }^{-1}∥=O_{P}\left(p_{n}^{2}/\sqrt{n}\right)$. Following the proof of Proposition 1 in [1], we can show that $∥{\hat{H}}_{n}-H_{n,\epsilon }∥=O_{P}\left(p_{n}^{2}/\sqrt{n}\right)$ and $∥{\hat{\Omega }}_{n}-\Omega_{n,\epsilon }∥=O_{P}\left(p_{n}^{2}/\sqrt{n}\right)$. To show $∥{\hat{H}}_{n}^{-1}{\hat{\Omega }}_{n}{\hat{H}}_{n}^{-1}-H_{n,\epsilon }^{-1}\Omega_{n,\epsilon }H_{n,\epsilon }^{-1}∥=O_{P}\left(p_{n}^{2}/\sqrt{n}\right)$, note ${\hat{H}}_{n}^{-1}{\hat{\Omega }}_{n}{\hat{H}}_{n}^{-1}-H_{n,\epsilon }^{-1}\Omega_{n,\epsilon }H_{n,\epsilon }^{-1}=L_{1}+L_{2}+L_{3}$, where $L_{1}={\hat{H}}_{n}^{-1}\left({\hat{\Omega }}_{n}-\Omega_{n,\epsilon }\right){\hat{H}}_{n}^{-1}$, $L_{2}={\hat{H}}_{n}^{-1}\left(H_{n,\epsilon }-{\hat{H}}_{n}\right)H_{n,\epsilon }^{-1}\Omega_{n,\epsilon }{\hat{H}}_{n}^{-1}$ and $L_{3}=H_{n,\epsilon }^{-1}\Omega_{n,\epsilon }{\hat{H}}_{n}^{-1}\left(H_{n,\epsilon }-{\hat{H}}_{n}\right)H_{n,\epsilon }^{-1}$. Following the proof in Lemma A2, it is straightforward to verify that $∥H_{n,\epsilon }^{-1}∥=O\left(1\right)$, $∥{\hat{H}}_{n}^{-1}∥=O_{P}\left(1\right)$. In addition, $∥H_{n,\epsilon }^{-1}\Omega_{n,\epsilon }∥=∥\left(H_{n,\epsilon }^{-1}-H_{n}^{-1}\right)\Omega_{n,\epsilon }+H_{n}^{-1}\left(\Omega_{n,\epsilon }-\Omega_{n}\right)+H_{n}^{-1}\Omega_{n}∥\leq ∥H_{n,\epsilon }^{-1}∥∥H_{n,\epsilon }-H_{n}∥∥H_{n}^{-1}∥∥\Omega_{n,\epsilon }∥+∥H_{n}^{-1}∥∥\Omega_{n,\epsilon }-\Omega_{n}∥+∥H_{n}^{-1}\Omega_{n}∥=O\left(1\right)$. Since $∥L_{1}∥\leq ∥{\hat{H}}_{n}^{-1}∥∥{\hat{\Omega }}_{n}-\Omega_{n,\epsilon }∥∥{\hat{H}}_{n}^{-1}∥$, we conclude $∥L_{1}∥=O_{P}\left(p_{n}^{2}/\sqrt{n}\right)$, and similarly $∥L_{2}∥=O_{P}\left(p_{n}^{2}/\sqrt{n}\right)$ and $∥L_{3}∥=O_{P}\left(p_{n}^{2}/\sqrt{n}\right)$. Hence, $∥{\hat{H}}_{n}^{-1}{\hat{\Omega }}_{n}{\hat{H}}_{n}^{-1}-H_{n,\epsilon }^{-1}\Omega_{n,\epsilon }H_{n,\epsilon }^{-1}∥=O_{P}\left(p_{n}^{2}/\sqrt{n}\right)$. Thus, we can conclude that $∥U\left(K_{n}\right)-U\left(K_{n,\epsilon }\right)∥=O_{P}\left(p_{n}^{2}/\sqrt{n}\right)$ and the eigenvalues of $U(K_{n})$ are uniformly bounded away from 0 and *∞* with probability tending to 1. Noting that $∥{\left\{U\left(K_{n}\right)\right\}}^{1/2}-{\left\{U\left(K_{n,\epsilon }\right)\right\}}^{1/2}{∥}^{2}\leq ∥U\left(K_{n}\right)-U\left(K_{n,\epsilon }\right)∥$. ☐

**Lemma** **A7** (asymptotic distribution of test statistic)**.** *Assume Conditions* A0–A9 *and* B4. *If $p_{n}^{6}/n\to 0$n→∞ and the distribution of $\left(X_{n},Y\right)$ is $K_{n,\epsilon }$, then*

$$\sqrt{n}\left[{\left\{U\left(K_{n}\right)\right\}}^{-1/2}A_{n}\left\{T\left(K_{n}\right)-{\tilde{\beta }}_{n,0}\right\}-U_{n}^{-1/2}A_{n}\left\{T(K_{n,\epsilon })-{\tilde{\beta }}_{n,0}\right\}\right]\overset{L}{⟶}N\left(0,I_{k}\right),$$

*where $A_{n}$ is any given $k\times (p_{n}+1)$ matrix such that $A_{n}A_{n}^{T}\to G$, with G being a k×k positive-definite matrix, and k is a fixed integer.*

**Proof.** Note that

$$\begin{array}{cc} & \sqrt{n}\left[{\left\{U\left(K_{n}\right)\right\}}^{-1/2}A_{n}\left\{T\left(K_{n}\right)-{\tilde{\beta }}_{n,0}\right\}-U_{n}^{-1/2}A_{n}\left\{T(K_{n,\epsilon })-{\tilde{\beta }}_{n,0}\right\}\right]\\ = & \sqrt{n}{\left\{U\left(K_{n}\right)\right\}}^{-1/2}A_{n}\left\{T\left(K_{n}\right)-T(K_{n,\epsilon })\right\}\\ & +\sqrt{n}\left[{\left\{U\left(K_{n}\right)\right\}}^{-1/2}-{\left\{U\left(K_{n,\epsilon }\right)\right\}}^{-1/2}\right]A_{n}\left\{T(K_{n,\epsilon })-{\tilde{\beta }}_{n,0}\right\}\\ & +\sqrt{n}\left[{\left\{U\left(K_{n,\epsilon }\right)\right\}}^{-1/2}-U_{n}^{-1/2}\right]A_{n}\left\{T(K_{n,\epsilon })-{\tilde{\beta }}_{n,0}\right\}\\ \equiv & I+\mathrm{II}+\mathrm{III}. \end{array}$$

For term I, we obtain from Lemma A4 that $\sqrt{n}{\left\{U\left(K_{n,\epsilon }\right)\right\}}^{-1/2}A_{n}\left(T\left(K_{n}\right)-T(K_{n,\epsilon })\right)\overset{L}{⟶}N\left(0,I_{k}\right).$ From Lemma A6, we get $∥{\left\{U\left(K_{n}\right)\right\}}^{-1/2}{\left\{U\left(K_{n,\epsilon }\right)\right\}}^{1/2}-I_{k}∥=o_{P}\left(1\right).$ Thus, by Slutsky theorem,

(A10) $$\begin{array}{c} I\overset{L}{⟶}N\left(0,I_{k}\right). \end{array}$$

For term II, we see from Lemma A6 that

$$\begin{array}{c} ∥{\left\{U\left(K_{n}\right)\right\}}^{-1/2}-{\left\{U\left(K_{n,\epsilon }\right)\right\}}^{-1/2}∥=O_{P}\left(p_{n}/n^{1/4}\right). \end{array}$$

Since

$$\begin{array}{c} ∥A_{n}\left\{T\left(K_{n,\epsilon }\right)-{\tilde{\beta }}_{n,0}\right\}∥\leq ∥A_{n}∥∥T(K_{n,\epsilon })-{\tilde{\beta }}_{n,0}∥=O\left(\sqrt{p_{n}/n}\right). \end{array}$$

Thus,

(A11) $$\begin{array}{c} ∥\mathrm{II}∥\leq \sqrt{n}∥{\left\{U\left(K_{n}\right)\right\}}^{-1/2}-{\left\{U\left(K_{n,\epsilon }\right)\right\}}^{-1/2}∥∥A_{n}∥∥T(K_{n,\epsilon })-{\tilde{\beta }}_{n,0}∥=O_{P}\left(p_{n}^{3/2}/n^{1/4}\right). \end{array}$$

Similarly, $∥\mathrm{III}∥=o_{P}\left(1\right).$ Combining (A10) and (A11) with Slutsky theorem completes the proof. ☐

**Lemma** **A8** (Influence Function IF)**.** *Assume Conditions* A1–A8 *and* B4. *For any fixed sample size n,*

$$\begin{array}{cc} & \frac{\partial }{\partial t}T\left(\left(1-t\right)K_{n,0}+tJ\right){|}_{t=t_{0}}\equiv \lim_{t\to t_{0}}\frac{T\left(\left(1-t\right)K_{n,0}+tJ\right)-T\left(\left(1-t_{0}\right)K_{n,0}+t_{0}J\right)}{t-t_{0}}\\ = & -H_{n,K_{t_{0}},T\left(K_{t_{0}}\right)}^{-1}\left[\;E_{J}\left\{\psi_{\mathrm{RBD}}\left(Z_{n};T\left(K_{t_{0}}\right)\right)\right\}-\;E_{K_{n,0}}\left\{\psi_{\mathrm{RBD}}\left(Z_{n};T\left(K_{t_{0}}\right)\right)\right\}\right], \end{array}$$

*where* $K_{t_{0}}=\left(1-t_{0}\right)K_{n,0}+t_{0}J$ *and* $t_{0}$ *is a positive constant such that* $t_{0}\leq c/p_{n}^{2}$ *with* c>0 *a sufficiently small constant. In addition,* $∥H_{n,K_{t_{0}},T\left(K_{t_{0}}\right)}^{-1}∥\leq C$ *uniformly for all n and* $t_{0}$ *such that* $t_{0}\leq c/p_{n}^{2}$ *with* c>0 *a sufficiently small constant.*

**Proof.** We follow the proof of Theorem 5.1 in [27]. Note

$$\begin{array}{cc} & \lim_{t\to t_{0}}\frac{T\left(\left(1-t\right)K_{n,0}+tJ\right)-T\left(\left(1-t_{0}\right)K_{n,0}+t_{0}J\right)}{t-t_{0}}\\ = & \lim_{\Delta \to 0}\frac{T\left(K_{t_{0}}+\Delta \left(J-K_{n,0}\right)\right)-T\left(K_{t_{0}}\right)}{\Delta }, \end{array}$$

where $\Delta =t-t_{0}$. It suffices to prove that for any sequence ${\left\{\Delta_{j}\right\}}_{j=1}^{\infty }$ such that $\lim_{j\to \infty }\Delta_{j}=0$, we have

$$\begin{array}{cc} & \lim_{j\to \infty }\frac{T\left(K_{t_{0}}+\Delta_{j}\left(J-K_{n,0}\right)\right)-T\left(K_{t_{0}}\right)}{\Delta_{j}}\\ = & -H_{n,K_{t_{0}},T\left(K_{t_{0}}\right)}^{-1}\left[\;E_{J}\left\{\psi_{\mathrm{RBD}}\left(Z_{n};T\left(K_{t_{0}}\right)\right)\right\}-\;E_{K_{n,0}}\left\{\psi_{\mathrm{RBD}}\left(Z_{n};T\left(K_{t_{0}}\right)\right)\right\}\right]. \end{array}$$

Following similar proofs in Lemma A1, we can show that for $t_{0}$ sufficiently small,

(A12) $$\begin{array}{c} ∥{\tilde{\beta }}_{n,0}-T\left(K_{t_{0}}\right)∥\leq Ct_{0}\sqrt{p_{n}}. \end{array}$$

Next we will show that the eigenvalues of $H_{n,K_{t_{0}},T\left(K_{t_{0}}\right)}$ are bounded away from 0.

$$\begin{array}{cc} & H_{n,K_{t_{0}},T\left(K_{t_{0}}\right)}=\left(1-t_{0}\right)H_{n,K_{n,0},T\left(K_{t_{0}}\right)}+t_{0}H_{n,J,T(K_{t_{0}})}\\ = & \left(1-t_{0}\right)H_{n}+t_{0}H_{n,J,{\tilde{\beta }}_{n,0}}+\left(1-t_{0}\right)\left\{H_{n,K_{n,0},T\left(K_{t_{0}}\right)}-H_{n}\right\}\\ & +t_{0}\left\{H_{n,J,T(K_{t_{0}})}-H_{n,J,{\tilde{\beta }}_{n,0}}\right\}=\left(1-t_{0}\right)I_{1}+t_{0}I_{2}+I_{3}+I_{4}. \end{array}$$

First,

$$\begin{array}{ccc} ∥I_{3}∥ & \leq & C\;E_{K_{n,0}}∥\left\{p_{2}\left(Y;{\tilde{X}}_{n}^{T}T\left(K_{t_{0}}\right)\right)-p_{2}\left(Y;{\tilde{X}}_{n}^{T}{\tilde{\beta }}_{n,0}\right)\right\}w\left(X_{n}\right){\tilde{X}}_{n}{\tilde{X}}_{n}^{T}∥\\ & \leq & Cp_{n}^{3/2}∥T\left(K_{t_{0}}\right)-{\tilde{\beta }}_{n,0}∥\leq Cp_{n}^{2}t_{0}. \end{array}$$

Similarly, $∥I_{2}∥\leq Cp_{n}t_{0}$ and $∥I_{4}∥\leq Cp_{n}^{2}t_{0}^{2}$. Since the eigenvalues of $I_{1}$ are bounded away from zero, $∥I_{2}∥$, $∥I_{3}∥$ and $∥I_{4}∥$ could be sufficiently small, we conclude that for $t_{0}\leq c/p_{n}^{2}$ when *c* is sufficiently small, the eigenvalues of $H_{n,K_{t_{0}},T\left(K_{t_{0}}\right)}$ are uniformly bounded away from 0. Define $K_{j}=K_{t_{0}}+\Delta_{j}\left(J-K_{n,0}\right)$. Following similar arguments for (A6) in Lemma A2, for *j* large enough, $\;E_{K_{j}}\left\{\psi_{\mathrm{RBD}}\left(Z_{n};T\left(K_{j}\right)\right)\right\}=0$. We will only consider *j* large enough below. The two term Taylor expansion yields

(A13) $$\begin{array}{c} 0=\;E_{K_{j}}\left\{\psi_{\mathrm{RBD}}\left(Z_{n};T\left(K_{j}\right)\right)\right\}=\;E_{K_{j}}\left\{\psi_{\mathrm{RBD}}\left(Z_{n};T\left(K_{t_{0}}\right)\right)\right\}+H_{n,K_{j},{\tilde{\beta }}_{j}^{*}}\left\{T\left(K_{j}\right)-T\left(K_{t_{0}}\right)\right\}, \end{array}$$

where ${\tilde{\beta }}_{j}^{*}$ lies between $T(K_{t_{0}})$ and $T(K_{j})$. Thus, from (A13) and the fact $\;E_{K_{j}}\left\{\psi_{\mathrm{RBD}}\left(Z_{n};T\left(K_{t_{0}}\right)\right)\right\}=\Delta_{j}\left[\;E_{J}\left\{\psi_{\mathrm{RBD}}\left(Z_{n};T\left(K_{t_{0}}\right)\right)\right\}-\;E_{K_{n,0}}\left\{\psi_{\mathrm{RBD}}\left(Z_{n};T\left(K_{t_{0}}\right)\right)\right\}\right]$, we have

$$\begin{array}{ccc} 0 & = & \;E_{K_{j}}\left\{\psi_{\mathrm{RBD}}\left(Z_{n};T\left(K_{t_{0}}\right)\right)\right\}+H_{n,K_{t_{0}},T\left(K_{t_{0}}\right)}\left\{T\left(K_{j}\right)-T\left(K_{t_{0}}\right)\right\}\\ & & +\left\{H_{n,K_{j},{\tilde{\beta }}_{j}^{*}}-H_{n,K_{t_{0}},T\left(K_{t_{0}}\right)}\right\}\left\{T\left(K_{j}\right)-T\left(K_{t_{0}}\right)\right\}\\ & = & \Delta_{j}\left[\;E_{J}\left\{\psi_{\mathrm{RBD}}\left(Z_{n};T\left(K_{t_{0}}\right)\right)\right\}-\;E_{K_{n,0}}\left\{\psi_{\mathrm{RBD}}\left(Z_{n};T\left(K_{t_{0}}\right)\right)\right\}\right]\\ & & +H_{n,K_{t_{0}},T\left(K_{t_{0}}\right)}\left\{T\left(K_{j}\right)-T\left(K_{t_{0}}\right)\right\}+\left(H_{n,K_{j},{\tilde{\beta }}_{j}^{*}}-H_{n,K_{t_{0}},T\left(K_{t_{0}}\right)}\right)\left\{T\left(K_{j}\right)-T\left(K_{t_{0}}\right)\right\}, \end{array}$$

and we obtain that

(A14) $$\begin{array}{cc} & T\left(K_{j}\right)-T\left(K_{t_{0}}\right)\\ = & -\Delta_{j}H_{n,K_{t_{0}},T\left(K_{t_{0}}\right)}^{-1}\left[\;E_{J}\left\{\psi_{\mathrm{RBD}}\left(Z_{n};T\left(K_{t_{0}}\right)\right)\right\}-\;E_{K_{n,0}}\left\{\psi_{\mathrm{RBD}}\left(Z_{n};T\left(K_{t_{0}}\right)\right)\right\}\right]\\ & -H_{n,K_{t_{0}},T\left(K_{t_{0}}\right)}^{-1}\left\{H_{n,K_{j},{\tilde{\beta }}_{j}^{*}}-H_{n,K_{t_{0}},T\left(K_{t_{0}}\right)}\right\}\left\{T\left(K_{j}\right)-T\left(K_{t_{0}}\right)\right\}. \end{array}$$

Next, we will show that $∥H_{n,K_{j},{\tilde{\beta }}_{j}^{*}}-H_{n,K_{t_{0}},T\left(K_{t_{0}}\right)}∥=o\left(1\right)$ as j→∞ for any fixed *n*. Since $∥{\tilde{\beta }}_{j}^{*}-T\left(K_{t_{0}}\right)∥\leq ∥T\left(K_{j}\right)-T\left(K_{t_{0}}\right)∥=O\left(\Delta_{j}\right)$,

(A15) $$\begin{array}{cc} & ∥H_{n,K_{j},{\tilde{\beta }}_{j}^{*}}-H_{n,K_{t_{0}},{\tilde{\beta }}_{j}^{*}}∥\\ = & \Delta_{j}∥\;E_{J}\left\{p_{2}\left(Y;{\tilde{X}}_{n}^{T}{\tilde{\beta }}_{j}^{*}\right)w\left(X_{n}\right){\tilde{X}}_{n}{\tilde{X}}_{n}^{T}\right\}-\;E_{K_{n,0}}\left\{p_{2}\left(Y;{\tilde{X}}_{n}^{T}{\tilde{\beta }}_{j}^{*}\right)w\left(X_{n}\right){\tilde{X}}_{n}{\tilde{X}}_{n}^{T}\right\}∥\\ = & O\left(\Delta_{j}\right)=o\left(1\right)\;\mathrm{as}\;j\to \infty , \end{array}$$

and also,

(A16) $$\begin{array}{cc} & ∥H_{n,K_{t_{0}},{\tilde{\beta }}_{j}^{*}}-H_{n,K_{t_{0}},T\left(K_{t_{0}}\right)}∥\\ = & ∥\;E_{K_{t_{0}}}\left[\left\{p_{2}\left(Y;{\tilde{X}}_{n}^{T}{\tilde{\beta }}_{j}^{*}\right)-p_{2}\left(Y;{\tilde{X}}_{n}^{T}T\left(K_{t_{0}}\right)\right)\right\}w\left(X_{n}\right){\tilde{X}}_{n}{\tilde{X}}_{n}^{T}\right]∥\\ = & o(1)\;\mathrm{as}\;j\to \infty . \end{array}$$

From Equations (A15) and (A16),

$$\begin{array}{c} ∥H_{n,K_{j},{\tilde{\beta }}_{j}^{*}}-H_{n,K_{t_{0}},T\left(K_{t_{0}}\right)}∥=o\left(1\right)\;\mathrm{as}\;j\to \infty \end{array}$$

which, together with Equations (A12) and (A14), implies that

$$\begin{array}{c} ∥T\left(K_{j}\right)-T\left(K_{t_{0}}\right)+\Delta_{j}H_{n,K_{t_{0}},T\left(K_{t_{0}}\right)}^{-1}\left[\;E_{J}\left\{\psi_{\mathrm{RBD}}\left(Z_{n};T\left(K_{t_{0}}\right)\right)\right\}-\;E_{K_{n,0}}\left\{\psi_{\mathrm{RBD}}\left(Z_{n};T\left(K_{t_{0}}\right)\right)\right\}\right]∥=o\left(\Delta_{j}\right). \end{array}$$

This completes the proof. ☐

**Lemma** **A9.** *Assume Conditions* A1–A8 *and* B4 *and* $\sup_{n}\;E_{J}(∥w\left(X_{n}\right){\tilde{X}}_{n}∥)\leq C$. *Let* $H_{k}\left(\cdot ;\delta \right)$ *be the cumulative distribution function of* $\chi_{k}^{2}\left(\delta \right)$ *distribution with δ the noncentrality parameter. Denote* $\delta \left(\epsilon \right)=n∥U_{n}^{-1/2}\left\{A_{n}T\left(K_{n,\epsilon }\right)-g_{0}\right\}{∥}^{2}$. *Let* $b\left(\epsilon \right)=-H_{k}\left(x;\delta \left(\epsilon \right)\right)$. *Then, for any fixed* x>0, $\sup_{\epsilon \in [0,C]}{lim sup}_{n\to \infty }\left|b^{\left(3\right)}\left(\epsilon \right)\right|\leq C$ *under* $H_{0}$ *and* $\sup_{\epsilon \in [0,C]}{lim sup}_{n\to \infty }\left|b^{\prime \prime }\left(\epsilon \right)\right|\leq C$ *under* $H_{1n}$.

**Proof.** Since $b\left(\epsilon \right)=-H_{k}\left(x;\delta \left(\epsilon \right)\right)$, we have

$$\begin{array}{ccc} b^{\prime }\left(\epsilon \right) & = & -\frac{\partial }{\partial \epsilon }H_{k}\left(x;\delta \left(\epsilon \right)\right)=\left\{-\frac{\partial }{\partial \delta }H_{k}\left(x;\delta \right){|}_{\delta =\delta (\epsilon )}\right\}\left\{\frac{\partial \delta (\epsilon )}{\partial \epsilon }\right\}\\ b^{\prime \prime }\left(\epsilon \right) & = & \left\{-\frac{\partial^{2}}{\partial \delta^{2}}H_{k}\left(x;\delta \right){|}_{\delta =\delta (\epsilon )}\right\}{\left\{\frac{\partial \delta (\epsilon )}{\partial \epsilon }\right\}}^{2}+\left\{-\frac{\partial }{\partial \delta }H_{k}\left(x;\delta \right){|}_{\delta =\delta (\epsilon )}\right\}\left\{\frac{\partial^{2}\delta \left(\epsilon \right)}{\partial \epsilon^{2}}\right\}\\ b^{\left(3\right)}\left(\epsilon \right) & = & \left\{-\frac{\partial^{3}}{\partial \delta^{3}}H_{k}\left(x;\delta \right){|}_{\delta =\delta (\epsilon )}\right\}{\left\{\frac{\partial \delta (\epsilon )}{\partial \epsilon }\right\}}^{3}\\ & & +3\left\{-\frac{\partial^{2}}{\partial \delta^{2}}H_{k}\left(x;\delta \right){|}_{\delta =\delta (\epsilon )}\right\}\left\{\frac{\partial \delta (\epsilon )}{\partial \epsilon }\right\}\left\{\frac{\partial^{2}\delta \left(\epsilon \right)}{\partial \epsilon^{2}}\right\}\\ & & +\left\{-\frac{\partial }{\partial \delta }H_{k}\left(x;\delta \right){|}_{\delta =\delta (\epsilon )}\right\}\left\{\frac{\partial^{3}\delta \left(\epsilon \right)}{\partial \epsilon^{3}}\right\}. \end{array}$$

To complete the proof, we only need to show that $\partial^{i}/\partial \delta^{i}H_{k}{\left(x;\delta \right)|}_{\delta =\delta (\epsilon )}$ and $\partial^{i}\delta \left(\epsilon \right)/\partial \epsilon^{i}$ (i=1,2,3) are bounded as n→∞ for all ϵ∈[0,C]. Note that

$$\begin{array}{c} H_{k}\left(x;\delta \right)=e^{-\delta /2}\sum_{j=0}^{\infty }\frac{{\left(\delta /2\right)}^{j}}{j!}\frac{\gamma (j+k/2,x/2)}{\Gamma (j+k/2)}, \end{array}$$

where Γ(·) is the Gamma function, and γ(·,·) is the lower incomplete gamma function $\gamma \left(s,x\right)=\int_{0}^{x}t^{s-1}e^{-t}dt,$ which satisfies $\gamma \left(s,x\right)=\left(s-1\right)\gamma \left(s-1,x\right)-x^{s-1}e^{-x}.$ Therefore,

$$\begin{array}{ccc} \frac{\partial }{\partial \delta }H_{k}\left(x;\delta \right) & = & -\frac{e^{-\delta /2}}{2}\sum_{j=0}^{\infty }\frac{{\left(\delta /2\right)}^{j}}{j!}\frac{\gamma (j+k/2,x/2)}{\Gamma (j+k/2)}+\frac{e^{-\delta /2}}{2}\sum_{j=1}^{\infty }\frac{{\left(\delta /2\right)}^{j-1}}{(j-1)!}\frac{\gamma (j+k/2,x/2)}{\Gamma (j+k/2)}\\ & = & \frac{1}{2}e^{-\delta /2}\sum_{j=0}^{\infty }\frac{{\left(\delta /2\right)}^{j}}{j!}\left\{-\frac{\gamma (j+k/2,x/2)}{\Gamma (j+k/2)}+\frac{\gamma (j+1+k/2,x/2)}{\Gamma (j+1+k/2)}\right\}. \end{array}$$

Since

$$\begin{array}{ccc} \frac{\gamma (j+1+k/2,x/2)}{\Gamma (j+1+k/2)} & = & \frac{\left(j+k/2\right)\gamma \left(j+k/2,x/2\right)-{\left(x/2\right)}^{j+k/2}e^{-x/2}}{\Gamma (j+1+k/2)}\\ & = & \frac{\gamma (j+k/2,x/2)}{\Gamma (j+k/2)}-\frac{{\left(x/2\right)}^{j+k/2}e^{-x/2}}{\Gamma (j+1+k/2)}, \end{array}$$

we have

$$\begin{array}{ccc} \frac{\partial }{\partial \delta }H_{k}\left(x;\delta \right) & = & -\frac{1}{2}e^{-\delta /2}\sum_{j=0}^{\infty }\frac{{\left(\delta /2\right)}^{j}}{j!}\frac{{\left(x/2\right)}^{j+k/2}e^{-x/2}}{\Gamma (j+1+k/2)}\\ \frac{\partial^{2}}{\partial \delta^{2}}H_{k}\left(x;\delta \right) & = & \frac{1}{4}e^{-\delta /2}\sum_{j=0}^{\infty }\frac{{\left(\delta /2\right)}^{j}}{j!}\frac{{\left(x/2\right)}^{j+k/2}e^{-x/2}}{\Gamma (j+1+k/2)}-\frac{1}{4}e^{-\delta /2}\sum_{j=0}^{\infty }\frac{{\left(\delta /2\right)}^{j}}{j!}\frac{{\left(x/2\right)}^{j+1+k/2}e^{-x/2}}{\Gamma (j+2+k/2)}\\ & = & \frac{1}{4}{\left(x/2\right)}^{k/2}e^{-x/2}e^{-\delta /2}\sum_{j=0}^{\infty }\frac{{\left(\delta /2\right)}^{j}}{j!}\left\{\frac{{\left(x/2\right)}^{j}}{\Gamma (j+1+k/2)}-\frac{{\left(x/2\right)}^{j+1}}{\Gamma (j+2+k/2)}\right\}\\ & = & \frac{1}{4}{\left(x/2\right)}^{k/2}e^{-x/2}e^{-\delta /2}\sum_{j=0}^{\infty }\frac{{\left(\delta /2\right)}^{j}}{j!}\frac{{\left(x/2\right)}^{j}}{\Gamma (j+1+k/2)}\left\{1-\frac{\left(x/2\right)}{j+1+k/2}\right\}\\ \frac{\partial^{3}}{\partial \delta^{3}}H_{k}\left(x;\delta \right) & = & -\frac{1}{8}{\left(x/2\right)}^{k/2}e^{-x/2}e^{-\delta /2}\sum_{j=0}^{\infty }\frac{{\left(\delta /2\right)}^{j}}{j!}\frac{{\left(x/2\right)}^{j}}{\Gamma (j+1+k/2)}\left\{1-\frac{\left(x/2\right)}{j+1+k/2}\right\}\\ & & +\frac{1}{8}{\left(x/2\right)}^{k/2}e^{-x/2}e^{-\delta /2}\sum_{j=0}^{\infty }\frac{{\left(\delta /2\right)}^{j}}{j!}\frac{{\left(x/2\right)}^{j+1}}{\Gamma (j+2+k/2)}\left\{1-\frac{\left(x/2\right)}{j+2+k/2}\right\}\\ & = & \frac{1}{8}{\left(x/2\right)}^{k/2}e^{-x/2}e^{-\delta /2}\sum_{j=0}^{\infty }\frac{{\left(\delta /2\right)}^{j}}{j!}\frac{{\left(x/2\right)}^{j}}{\Gamma (j+1+k/2)}\\ & & \cdot \left[\frac{\left(x/2\right)}{j+1+k/2}\left\{1-\frac{\left(x/2\right)}{j+2+k/2}\right\}-\left\{1-\frac{\left(x/2\right)}{j+1+k/2}\right\}\right]. \end{array}$$

From the results of Lemma A3, that |δ(ϵ)| is bounded as n→∞ for all ϵ∈[0,C] under both $H_{0}$ and $H_{1n}$, so are $\partial^{i}/\partial \delta^{i}H_{k}{\left(x;\delta \right)|}_{\delta =\delta (\epsilon )}$ (i=1,2,3). Now, we consider the derivatives of δ(ϵ),

$$\begin{array}{ccc} \frac{\partial \delta (\epsilon )}{\partial \epsilon } & = & 2n{\left\{A_{n}\frac{\partial T(K_{n,\epsilon })}{\partial \epsilon }\right\}}^{T}U_{n}^{-1}\left\{A_{n}T\left(K_{n,\epsilon }\right)-g_{0}\right\}\\ \frac{\partial^{2}\delta \left(\epsilon \right)}{\partial \epsilon^{2}} & = & 2n{\left\{A_{n}\frac{\partial T(K_{n,\epsilon })}{\partial \epsilon }\right\}}^{T}U_{n}^{-1}\left\{A_{n}\frac{\partial T(K_{n,\epsilon })}{\partial \epsilon }\right\}\\ & & +2n{\left\{A_{n}\frac{\partial^{2}T\left(K_{n,\epsilon }\right)}{\partial \epsilon^{2}}\right\}}^{T}U_{n}^{-1}\left\{A_{n}T\left(K_{n,\epsilon }\right)-g_{0}\right\}\\ \frac{\partial^{3}\delta \left(\epsilon \right)}{\partial \epsilon^{3}} & = & 6n{\left\{A_{n}\frac{\partial^{2}T\left(K_{n,\epsilon }\right)}{\partial \epsilon^{2}}\right\}}^{T}U_{n}^{-1}\left\{A_{n}\frac{\partial T(K_{n,\epsilon })}{\partial \epsilon }\right\}\\ & & +2n{\left\{A_{n}\frac{\partial^{3}T\left(K_{n,\epsilon }\right)}{\partial \epsilon^{3}}\right\}}^{T}U_{n}^{-1}\left\{A_{n}T\left(K_{n,\epsilon }\right)-g_{0}\right\}. \end{array}$$

To complete the proof, we only need to show that $\sqrt{n}∥\partial^{i}/\partial \epsilon^{i}T\left(K_{n,\epsilon }\right)∥$ (i=1,2,3) are bounded as n→∞ for all ϵ∈[0,C], and $\sqrt{n}∥A_{n}T\left(K_{n,\epsilon }\right)-g_{0}∥$ is bounded under $H_{0}$ and $H_{1n}$ as n→∞ for all ϵ∈[0,C]. The result for $\sqrt{n}∥A_{n}T\left(K_{n,\epsilon }\right)-g_{0}∥$ is straightforward from Lemma A3. First, for the first order derivative of $T(K_{n,\epsilon })$,

$$\begin{array}{cc} & \sqrt{n}\frac{\partial }{\partial \epsilon }T\left(K_{n,\epsilon }\right)\\ = & -H_{n,K_{n,\epsilon },T\left(K_{n,\epsilon }\right)}^{-1}\left[\;E_{J}\left\{p_{1}\left(Y;{\tilde{X}}_{n}^{T}T\left(K_{n,\epsilon }\right)\right)w\left(X_{n}\right){\tilde{X}}_{n}\right\}-\;E_{K_{n,0}}\left\{p_{1}\left(Y;{\tilde{X}}_{n}^{T}T\left(K_{n,\epsilon }\right)\right)w\left(X_{n}\right){\tilde{X}}_{n}\right\}\right]. \end{array}$$

Since $∥H_{n,K_{n,\epsilon },T\left(K_{n,\epsilon }\right)}^{-1}∥\leq C$, $∥\;E_{J}\left\{p_{1}\left(Y;{\tilde{X}}_{n}^{T}T\left(K_{n,\epsilon }\right)\right)w\left(X_{n}\right){\tilde{X}}_{n}\right\}∥\leq C\;E_{J}∥w\left(X_{n}\right){\tilde{X}}_{n}∥\leq C$ and

$$\begin{array}{cc} & ∥\;E_{K_{n,0}}\left\{p_{1}\left(Y;{\tilde{X}}_{n}^{T}T\left(K_{n,\epsilon }\right)\right)w\left(X_{n}\right){\tilde{X}}_{n}\right\}∥\\ = & ∥\;E_{K_{n,0}}\left\{p_{1}\left(Y;{\tilde{X}}_{n}^{T}T\left(K_{n,\epsilon }\right)\right)w\left(X_{n}\right){\tilde{X}}_{n}\right\}-\;E_{K_{n,0}}\left\{p_{1}\left(Y;{\tilde{X}}_{n}^{T}{\tilde{\beta }}_{n,0}\right)w\left(X_{n}\right){\tilde{X}}_{n}\right\}∥\\ = & ∥\;E_{K_{n,0}}\left[p_{2}\left(Y;{\tilde{X}}_{n}^{T}{\tilde{\beta }}^{*}\right)w\left(X_{n}\right){\tilde{X}}_{n}{\tilde{X}}_{n}^{T}\left\{T\left(K_{n,\epsilon }\right)-{\tilde{\beta }}_{n,0}\right\}\right]∥\\ \leq & Cp_{n}^{3/2}/\sqrt{n}, \end{array}$$

we conclude that $\sqrt{n}∥\partial /\partial \epsilon T\left(K_{n,\epsilon }\right)∥$ is uniformly bounded for all ϵ∈[0,C] as n→∞. Second, for the second order derivative of $T(K_{n,\epsilon })$,

$$\begin{array}{cc} & \sqrt{n}\frac{\partial^{2}}{\partial \epsilon^{2}}T\left(\left(1-\epsilon /\sqrt{n}\right)K_{n,0}+\epsilon /\sqrt{n}J\right)\\ = & -\frac{\partial H_{n,K_{n,\epsilon },T\left(K_{n,\epsilon }\right)}^{-1}}{\partial \epsilon }\\ & \cdot [\;E_{J}\left\{p_{1}\left(Y;{\tilde{X}}_{n}^{T}T\left(K_{n,\epsilon }\right)\right)w\left(X_{n}\right){\tilde{X}}_{n}\right\}-\;E_{K_{n,0}}\left\{p_{1}\left(Y;{\tilde{X}}_{n}^{T}T\left(K_{n,\epsilon }\right)\right)w\left(X_{n}\right){\tilde{X}}_{n}\right\}]\\ & -H_{n,K_{n,\epsilon },T\left(K_{n,\epsilon }\right)}^{-1}\\ & \cdot \frac{\partial }{\partial \epsilon }\left[\;E_{J}\left\{p_{1}\left(Y;{\tilde{X}}_{n}^{T}T\left(K_{n,\epsilon }\right)\right)w\left(X_{n}\right){\tilde{X}}_{n}\right\}-\;E_{K_{n,0}}\left\{p_{1}\left(Y;{\tilde{X}}_{n}^{T}T\left(K_{n,\epsilon }\right)\right)w\left(X_{n}\right){\tilde{X}}_{n}\right\}\right] \end{array}$$

with

$$\begin{array}{ccc} \frac{\partial }{\partial \epsilon }H_{n,K_{n,\epsilon },T\left(K_{n,\epsilon }\right)}^{-1} & = & -H_{n,K_{n,\epsilon },T\left(K_{n,\epsilon }\right)}^{-1}\frac{\partial H_{n,K_{n,\epsilon },T\left(K_{n,\epsilon }\right)}}{\partial \epsilon }H_{n,K_{n,\epsilon },T\left(K_{n,\epsilon }\right)}^{-1},\\ \frac{\partial H_{n,K_{n,\epsilon },T\left(K_{n,\epsilon }\right)}}{\partial \epsilon } & = & -\frac{1}{\sqrt{n}}\;E_{K_{n,0}}\left\{p_{2}\left(Y;{\tilde{X}}_{n}^{T}T\left(K_{n,\epsilon }\right)\right)w\left(X_{n}\right){\tilde{X}}_{n}{\tilde{X}}_{n}^{T}\right\}\\ & & +\frac{1}{\sqrt{n}}\;E_{J}\left\{p_{2}\left(Y;{\tilde{X}}_{n}^{T}T\left(K_{n,\epsilon }\right)\right)w\left(X_{n}\right){\tilde{X}}_{n}{\tilde{X}}_{n}^{T}\right\}\\ & & +\left(1-\epsilon /\sqrt{n}\right)\;E_{K_{n,0}}\left\{p_{3}\left(Y;{\tilde{X}}_{n}^{T}T\left(K_{n,\epsilon }\right)\right)w\left(X_{n}\right){\tilde{X}}_{n}{\tilde{X}}_{n}^{T}{\tilde{X}}_{n}^{T}\frac{\partial }{\partial \epsilon }T\left(K_{n,\epsilon }\right)\right\}\\ & & +\epsilon /\sqrt{n}\;E_{J}\left\{p_{3}\left(Y;{\tilde{X}}_{n}^{T}T\left(K_{n,\epsilon }\right)\right)w\left(X_{n}\right){\tilde{X}}_{n}{\tilde{X}}_{n}^{T}{\tilde{X}}_{n}^{T}\frac{\partial }{\partial \epsilon }T\left(K_{n,\epsilon }\right)\right\}. \end{array}$$

Therefore, $∥\partial /\partial \epsilon H_{n,K_{n,\epsilon },T\left(K_{n,\epsilon }\right)}^{-1}∥\leq C∥\partial /\partial \epsilon H_{n,K_{n,\epsilon },T\left(K_{n,\epsilon }\right)}∥\leq Cp_{n}^{3/2}/\sqrt{n}$. In addition,

$$\begin{array}{cc} & ∥\frac{\partial }{\partial \epsilon }\left[\;E_{J}\left\{p_{1}\left(Y;{\tilde{X}}_{n}^{T}T\left(K_{n,\epsilon }\right)\right)w\left(X_{n}\right){\tilde{X}}_{n}\right\}-\;E_{K_{n,0}}\left\{p_{1}\left(Y;{\tilde{X}}_{n}^{T}T\left(K_{n,\epsilon }\right)\right)w\left(X_{n}\right){\tilde{X}}_{n}\right\}\right]∥\\ = & ∥\;E_{J}\left\{p_{2}\left(Y;{\tilde{X}}_{n}^{T}T\left(K_{n,\epsilon }\right)\right)w\left(X_{n}\right){\tilde{X}}_{n}{\tilde{X}}_{n}^{T}\frac{\partial }{\partial \epsilon }T\left(K_{n,\epsilon }\right)\right\}\\ & -\;E_{K_{n,0}}\left\{p_{2}\left(Y;{\tilde{X}}_{n}^{T}T\left(K_{n,\epsilon }\right)\right)w\left(X_{n}\right){\tilde{X}}_{n}{\tilde{X}}_{n}^{T}\frac{\partial }{\partial \epsilon }T\left(K_{n,\epsilon }\right)\right\}∥\\ \leq & Cp_{n}/\sqrt{n}. \end{array}$$

Therefore, $∥\sqrt{n}\frac{\partial^{2}}{\partial \epsilon^{2}}T\left(\left(1-\epsilon /\sqrt{n}\right)K_{n,0}+\epsilon /\sqrt{n}J\right)∥=o\left(1\right)$ for all ϵ∈[0,C]. Finally, for the third order derivative of $T(K_{n,\epsilon })$,

$$\begin{array}{cc} & \sqrt{n}\frac{\partial^{3}}{\partial \epsilon^{3}}T\left(\left(1-\epsilon /\sqrt{n}\right)K_{n,0}+\epsilon /\sqrt{n}J\right)\\ = & -\frac{\partial^{2}H_{n,K_{n,\epsilon },T\left(K_{n,\epsilon }\right)}^{-1}}{\partial \epsilon^{2}}\\ & \cdot [\;E_{J}\left\{p_{1}\left(Y;{\tilde{X}}_{n}^{T}T\left(K_{n,\epsilon }\right)\right)w\left(X_{n}\right){\tilde{X}}_{n}\right\}-\;E_{K_{n,0}}\left\{p_{1}\left(Y;{\tilde{X}}_{n}^{T}T\left(K_{n,\epsilon }\right)\right)w\left(X_{n}\right){\tilde{X}}_{n}\right\}]\\ & -2\frac{\partial H_{n,K_{n,\epsilon },T\left(K_{n,\epsilon }\right)}^{-1}}{\partial \epsilon }\\ & \cdot \frac{\partial }{\partial \epsilon }\left[\;E_{J}\left\{p_{1}\left(Y;{\tilde{X}}_{n}^{T}T\left(K_{n,\epsilon }\right)\right)w\left(X_{n}\right){\tilde{X}}_{n}\right\}-\;E_{K_{n,0}}\left\{p_{1}\left(Y;{\tilde{X}}_{n}^{T}T\left(K_{n,\epsilon }\right)\right)w\left(X_{n}\right){\tilde{X}}_{n}\right\}\right]\\ & -H_{n,K_{n,\epsilon },T\left(K_{n,\epsilon }\right)}^{-1}\\ & \cdot \frac{\partial^{2}}{\partial \epsilon^{2}}\left[\;E_{J}\left\{p_{1}\left(Y;{\tilde{X}}_{n}^{T}T\left(K_{n,\epsilon }\right)\right)w\left(X_{n}\right){\tilde{X}}_{n}\right\}-\;E_{K_{n,0}}\left\{p_{1}\left(Y;{\tilde{X}}_{n}^{T}T\left(K_{n,\epsilon }\right)\right)w\left(X_{n}\right){\tilde{X}}_{n}\right\}\right]. \end{array}$$

Note:

$$\begin{array}{cc} & \frac{\partial^{2}}{\partial \epsilon^{2}}H_{n,K_{n,\epsilon },T\left(K_{n,\epsilon }\right)}^{-1}\\ = & -\frac{\partial H_{n,K_{n,\epsilon },T\left(K_{n,\epsilon }\right)}^{-1}}{\partial \epsilon }\frac{\partial H_{n,K_{n,\epsilon },T\left(K_{n,\epsilon }\right)}}{\partial \epsilon }H_{n,K_{n,\epsilon },T\left(K_{n,\epsilon }\right)}^{-1}\\ & -H_{n,K_{n,\epsilon },T\left(K_{n,\epsilon }\right)}^{-1}\frac{\partial^{2}H_{n,K_{n,\epsilon },T\left(K_{n,\epsilon }\right)}}{\partial \epsilon^{2}}H_{n,K_{n,\epsilon },T\left(K_{n,\epsilon }\right)}^{-1}\\ & -H_{n,K_{n,\epsilon },T\left(K_{n,\epsilon }\right)}^{-1}\frac{\partial H_{n,K_{n,\epsilon },T\left(K_{n,\epsilon }\right)}}{\partial \epsilon }\frac{\partial H_{n,K_{n,\epsilon },T\left(K_{n,\epsilon }\right)}^{-1}}{\partial \epsilon }, \end{array}$$

where

$$\begin{array}{cc} & \frac{\partial^{2}}{\partial \epsilon^{2}}H_{n,K_{n,\epsilon },T\left(K_{n,\epsilon }\right)}\\ = & -\frac{2}{\sqrt{n}}\;E_{K_{n,0}}\left\{p_{3}\left(Y;{\tilde{X}}_{n}^{T}T\left(K_{n,\epsilon }\right)\right)w\left(X_{n}\right){\tilde{X}}_{n}{\tilde{X}}_{n}^{T}{\tilde{X}}_{n}^{T}\frac{\partial }{\partial \epsilon }T\left(K_{n,\epsilon }\right)\right\}\\ & +\frac{2}{\sqrt{n}}\;E_{J}\left\{p_{3}\left(Y;{\tilde{X}}_{n}^{T}T\left(K_{n,\epsilon }\right)\right)w\left(X_{n}\right){\tilde{X}}_{n}{\tilde{X}}_{n}^{T}{\tilde{X}}_{n}^{T}\frac{\partial }{\partial \epsilon }T\left(K_{n,\epsilon }\right)\right\}\\ & +\left(1-\epsilon /\sqrt{n}\right)\;E_{K_{n,0}}\left\{p_{4}\left(Y;{\tilde{X}}_{n}^{T}T\left(K_{n,\epsilon }\right)\right)w\left(X_{n}\right){\tilde{X}}_{n}{\tilde{X}}_{n}^{T}{\left({\tilde{X}}_{n}^{T}\frac{\partial }{\partial \epsilon }T\left(K_{n,\epsilon }\right)\right)}^{2}\right\}\\ & +\epsilon /\sqrt{n}\;E_{J}\left\{p_{4}\left(Y;{\tilde{X}}_{n}^{T}T\left(K_{n,\epsilon }\right)\right)w\left(X_{n}\right){\tilde{X}}_{n}{\tilde{X}}_{n}^{T}{\left({\tilde{X}}_{n}^{T}\frac{\partial }{\partial \epsilon }T\left(K_{n,\epsilon }\right)\right)}^{2}\right\}. \end{array}$$

Hence, $∥\frac{\partial^{2}}{\partial \epsilon^{2}}H_{n,K_{n,\epsilon },T\left(K_{n,\epsilon }\right)}∥\leq Cp_{n}^{2}/n$ which implies that $∥\frac{\partial^{2}}{\partial \epsilon^{2}}H_{n,K_{n,\epsilon },T\left(K_{n,\epsilon }\right)}^{-1}∥=o\left(1\right)$ for all ϵ∈[0,C]. In addition,

$$\begin{array}{cc} & \frac{\partial^{2}}{\partial \epsilon^{2}}\left[\;E_{J}\left\{p_{1}\left(Y;{\tilde{X}}_{n}^{T}T\left(K_{n,\epsilon }\right)\right)w\left(X_{n}\right){\tilde{X}}_{n}\right\}-\;E_{K_{n,0}}\left\{p_{1}\left(Y;{\tilde{X}}_{n}^{T}T\left(K_{n,\epsilon }\right)\right)w\left(X_{n}\right){\tilde{X}}_{n}\right\}\right]\\ = & \frac{\partial }{\partial \epsilon }[\;E_{J}\left\{p_{2}\left(Y;{\tilde{X}}_{n}^{T}T\left(K_{n,\epsilon }\right)\right)w\left(X_{n}\right){\tilde{X}}_{n}{\tilde{X}}_{n}^{T}\frac{\partial }{\partial \epsilon }T\left(K_{n,\epsilon }\right)\right\}\\ & -\;E_{K_{n,0}}\left\{p_{2}\left(Y;{\tilde{X}}_{n}^{T}T\left(K_{n,\epsilon }\right)\right)w\left(X_{n}\right){\tilde{X}}_{n}{\tilde{X}}_{n}^{T}\frac{\partial }{\partial \epsilon }T\left(K_{n,\epsilon }\right)\right\}]\\ = & \;E_{J}\left\{p_{3}\left(Y;{\tilde{X}}_{n}^{T}T\left(K_{n,\epsilon }\right)\right)w\left(X_{n}\right){\tilde{X}}_{n}{\left({\tilde{X}}_{n}^{T}\frac{\partial }{\partial \epsilon }T\left(K_{n,\epsilon }\right)\right)}^{2}\right\}\\ & +\;E_{J}\left\{p_{2}\left(Y;{\tilde{X}}_{n}^{T}T\left(K_{n,\epsilon }\right)\right)w\left(X_{n}\right){\tilde{X}}_{n}{\tilde{X}}_{n}^{T}\frac{\partial^{2}}{\partial \epsilon^{2}}T\left(K_{n,\epsilon }\right)\right\}\\ & -\;E_{{\tilde{\beta }}_{n,0}}\left\{p_{3}\left(Y;{\tilde{X}}_{n}^{T}T\left(K_{n,\epsilon }\right)\right)w\left(X_{n}\right){\tilde{X}}_{n}{\left({\tilde{X}}_{n}^{T}\frac{\partial }{\partial \epsilon }T\left(K_{n,\epsilon }\right)\right)}^{2}\right\}\\ & -\;E_{{\tilde{\beta }}_{n,0}}\left\{p_{2}\left(Y;{\tilde{X}}_{n}^{T}T\left(K_{n,\epsilon }\right)\right)w\left(X_{n}\right){\tilde{X}}_{n}{\tilde{X}}_{n}^{T}\frac{\partial^{2}}{\partial \epsilon^{2}}T\left(K_{n,\epsilon }\right)\right\}. \end{array}$$

Hence, $∥\frac{\partial^{2}}{\partial \epsilon^{2}}\left[\;E_{J}\left\{p_{1}\left(Y;{\tilde{X}}_{n}^{T}T\left(K_{n,\epsilon }\right)\right)w\left(X_{n}\right){\tilde{X}}_{n}\right\}-\;E_{K_{n,0}}\left\{p_{1}\left(Y;{\tilde{X}}_{n}^{T}T\left(K_{n,\epsilon }\right)\right)w\left(X_{n}\right){\tilde{X}}_{n}\right\}\right]∥\leq Cp_{n}/\sqrt{n}$. Therefore, $∥\sqrt{n}\frac{\partial^{3}}{\partial \epsilon^{3}}T\left(\left(1-\epsilon /\sqrt{n}\right)K_{n,0}+\epsilon /\sqrt{n}J\right)∥=o\left(1\right)$ for all ϵ∈[0,C]. Hence, we complete the proof. ☐

**Proof** **of** **Theorem** **1**. We follow the idea of the proof in [10]. Lemma A7 implies that the Wald-type test statistic $W_{n}$ is asymptotically noncentral $\chi_{k}^{2}$ with noncentrality parameter $\delta \left(\epsilon \right)=n∥U_{n}^{-1/2}\left\{A_{n}T\left(K_{n,\epsilon }\right)-g_{0}\right\}{∥}^{2}$. Therefore, $\alpha \left(K_{n,\epsilon }\right)=P\left(W_{n}>\eta_{{}_{1-\alpha_{{}_{0}}}}|H_{0}\right)=1-H_{k}\left(\eta_{{}_{1-\alpha_{{}_{0}}}};\delta \left(\epsilon \right)\right)+h\left(n,\epsilon \right)$ where $h\left(n,\epsilon \right)=\alpha \left(K_{n,\epsilon }\right)-1+H_{k}\left(\eta_{{}_{1-\alpha_{{}_{0}}}};\delta \left(\epsilon \right)\right)\to 0$ as n→∞ for any fixed ϵ. Let $b\left(\epsilon \right)=-H_{k}\left(\eta_{{}_{1-\alpha_{{}_{0}}}};\delta \left(\epsilon \right)\right)$. Then, for ϵ close to 0, we have

(A17) $$\begin{array}{cc} & \alpha \left(K_{n,\epsilon }\right)-\alpha_{{}_{0}}=b\left(\epsilon \right)-b\left(0\right)+h\left(n,\epsilon \right)-h\left(n,0\right)\\ = & \epsilon b^{\prime }\left(0\right)+\frac{1}{2}\epsilon^{2}b^{\prime \prime }\left(0\right)+\frac{1}{6}\epsilon^{3}b^{\left(3\right)}\left(\epsilon^{*}\right)+h\left(n,\epsilon \right)-h\left(n,0\right), \end{array}$$

where $0<\epsilon^{*}<\epsilon$. Note that under $H_{0}$

$$\begin{array}{c} b^{\prime }\left(0\right)=\mu_{k}\left\{\frac{\partial \delta (\epsilon )}{\partial \epsilon }{|}_{\epsilon =0}\right\}=2\mu_{k}n{\left\{A_{n}\frac{\partial T(K_{n,\epsilon })}{\partial \epsilon }\right\}}^{T}{|}_{\epsilon =0}U_{n}^{-1}\left\{A_{n}{\tilde{\beta }}_{n,0}-g_{0}\right\}=0. \end{array}$$

From Lemma A8, under $H_{0}$

$$\begin{array}{c} \frac{\partial T(K_{n,\epsilon })}{\partial \epsilon }{|}_{\epsilon =0}=1/\sqrt{n}\;E_{J}\left\{\mathrm{IF}\left(Z_{n};T,K_{n,0}\right)\right\}. \end{array}$$

Thus,

$$\begin{array}{ccc} b^{\prime \prime }\left(0\right) & = & \mu_{k}\left\{\frac{\partial^{2}\delta \left(\epsilon \right)}{\partial \epsilon^{2}}{|}_{\epsilon =0}\right\}=2\mu_{k}{∥U_{n}^{-1/2}A_{n}\;E_{J}\left\{\mathrm{IF}\left(Z_{n};T,K_{n,0}\right)\right\}∥}^{2}. \end{array}$$

Since from Lemma A8, $\mathrm{IF}\left(z_{n};T,K_{n,0}\right)=-H_{n}^{-1}\;E_{J}\left\{\psi_{\mathrm{RBD}}\left(z_{n};{\tilde{\beta }}_{n,0}\right)\right\}$ is uniformly bounded, we have

$$\begin{array}{c} D=\underset{n\to \infty }{lim sup}{∥U_{n}^{-1/2}A_{n}\;E_{J}\left\{\mathrm{IF}\left(Z_{n};T,K_{n,0}\right)\right\}∥}^{2}<\infty . \end{array}$$

From Equation (A17)

$$\begin{array}{c} \underset{n\to \infty }{lim sup}\alpha \left(K_{n,\epsilon }\right)=\alpha_{{}_{0}}+\epsilon^{2}\mu_{k}D+o\left(\epsilon^{2}\right), \end{array}$$

since $\sup_{\epsilon \in [0,C]}{lim sup}_{n\to \infty }\left|b^{\left(3\right)}\left(\epsilon \right)\right|\leq C$ from Lemma A9. We complete the proof. ☐

**Proof** **of** **Corollary** **1.** For Part (i), following the proof of Theorem 1, for any fixed z,

$$\lim_{n\to \infty }\alpha \left(K_{n,\epsilon }\right)=\alpha_{{}_{0}}+\epsilon^{2}\mu_{k}{∥U^{-1/2}A\;\mathrm{IF}\left(z;T,K_{0}\right)∥}^{2}+d\left(z,\epsilon \right),$$

where $d\left(z,\epsilon \right)=o\left(\epsilon^{2}\right)$. From the assumption that $\sup_{x\in R^{p}}∥w\left(x\right)x∥\leq C$ and $\sup_{\mu \in R}\left|q^{\prime \prime }\left(\mu \right)\sqrt{V(\mu )}/F^{\prime }\left(\mu \right)\right|\leq C$, we know $D_{1}\leq \infty$. Following the proof of Lemma A9, $\sup_{z\in R}\left|d\left(z,\epsilon \right)\right|=o\left(\epsilon^{2}\right)$. We finished the proof of part (i). Part (ii) is straightforward by applying Theorem 1 with $J=\Delta_{z_{n}}$. ☐

**Proof** **of** **Theorem** **2.** Lemma A7 implies that

$$\begin{array}{c} \sqrt{n}[{\left\{U\left(K_{n}\right)\right\}}^{-1/2}\left\{A_{n}T\left(K_{n}\right)-g_{0}\right\}-{\left\{U\left(K_{n}\right)\right\}}^{-1/2}\left(A_{n}{\tilde{\beta }}_{n,0}-g_{0}\right)\\ \;-U_{n}^{-1/2}A_{n}\left\{T(K_{n,\epsilon })-{\tilde{\beta }}_{n,0}\right\}]\overset{L}{⟶}\left(0,I_{k}\right). \end{array}$$

From Lemmas A5 and A6,

$$\begin{array}{c} \sqrt{n}\left[{\left\{U\left(K_{n}\right)\right\}}^{-1/2}\left\{A_{n}T\left(K_{n}\right)-g_{0}\right\}-U_{n}^{-1/2}\left\{A_{n}T(K_{n,\epsilon })-g_{0}\right\}\right]\overset{L}{⟶}\left(0,I_{k}\right). \end{array}$$

Then, $W_{n}$ is asymptotically $\chi_{k}^{2}\left(\delta \left(\epsilon \right)\right)$ with $\delta \left(\epsilon \right)=n∥U_{n}^{-1/2}\left\{A_{n}T\left(K_{n,\epsilon }\right)-g_{0}\right\}{∥}^{2}$ under $H_{1n}$. Therefore, $\beta \left(K_{n,\epsilon }\right)=P\left(W_{n}>\eta_{{}_{1-\alpha_{{}_{0}}}}|H_{1n}\right)=1-H_{k}\left(\eta_{{}_{1-\alpha_{{}_{0}}}};\delta \left(\epsilon \right)\right)+h\left(n,\epsilon \right)$, where $h\left(n,\epsilon \right)=\beta \left(K_{n,\epsilon }\right)-1+H_{k}\left(\eta_{{}_{1-\alpha_{{}_{0}}}};\delta \left(\epsilon \right)\right)\to 0$ as n→∞ for any fixed ϵ. Let $b\left(\epsilon \right)=-H_{k}\left(\eta_{{}_{1-\alpha_{{}_{0}}}};\delta \left(\epsilon \right)\right)$. Then, for ϵ close to 0, we have

(A18) $$\begin{array}{cc} & \beta \left(K_{n,\epsilon }\right)-\beta_{0}=b\left(\epsilon \right)-b\left(0\right)+h\left(n,\epsilon \right)-h\left(n,0\right)\\ = & \epsilon b^{\prime }\left(0\right)+\frac{1}{2}\epsilon^{2}b^{\prime \prime }\left(\epsilon^{*}\right)+h\left(n,\epsilon \right)-h\left(n,0\right), \end{array}$$

where $0<\epsilon^{*}<\epsilon$. Note that under $H_{1n}$, $\delta \left(0\right)=n∥U_{n}^{-1/2}\left(A_{n}{\tilde{\beta }}_{n,0}-g_{0}\right){∥}^{2}=c^{T}U_{n}^{-1}c.$ Then,

$$\begin{array}{ccc} b^{\prime }\left(0\right) & = & \frac{-\partial H_{k}\left(\eta_{{}_{1-\alpha_{{}_{0}}}};\delta \right)}{\partial \delta }{|}_{\delta =\delta (0)}\frac{\partial \delta (\epsilon )}{\partial \epsilon }{|}_{\epsilon =0}=2\nu_{k}n{\left\{A_{n}\frac{\partial T(K_{n,\epsilon })}{\partial \epsilon }\right\}}^{T}{|}_{\epsilon =0}U_{n}^{-1}\left\{A_{n}{\tilde{\beta }}_{n,0}-g_{0}\right\}\\ & = & 2\nu_{k}\sqrt{n}{\left\{A_{n}\frac{\partial T(K_{n,\epsilon })}{\partial \epsilon }\right\}}^{T}{|}_{\epsilon =0}U_{n}^{-1}c. \end{array}$$

From Lemma A8,

$$\begin{array}{c} \frac{\partial T(K_{n,\epsilon })}{\partial \epsilon }{|}_{\epsilon =0}=1/\sqrt{n}\;E_{J}\left\{\mathrm{IF}\left(Z_{n};T,K_{n,0}\right)\right\}, \end{array}$$

and hence,

$$\begin{array}{c} b^{\prime }\left(0\right)=2\nu_{k}c^{T}U_{n}^{-1}A_{n}\;E_{J}\left\{\mathrm{IF}\left(Z_{n};T,K_{n,0}\right)\right\}. \end{array}$$

Since $\sup_{\epsilon \in [0,C]}{lim sup}_{n\to \infty }\left|b^{\prime \prime }\left(\epsilon \right)\right|\leq C$ under $H_{1n}$ by Lemma A9, we have ${lim inf}_{n\to \infty }1/2\epsilon^{2}b^{\prime \prime }\left(\epsilon^{*}\right)=o\left(\epsilon \right)$ as ϵ→0. Since from Lemma A8, $\mathrm{IF}\left(z_{n};T,K_{n,0}\right)=-H_{n}^{-1}\;E_{J}\left\{\psi_{\mathrm{RBD}}\left(z_{n};{\tilde{\beta }}_{n,0}\right)\right\}$ is uniformly bounded,

$$\begin{array}{c} \left|B|=\right|\underset{n\to \infty }{lim inf}2c^{T}U_{n}^{-1}A_{n}\;E_{J}\left\{\mathrm{IF}\left(Z_{n};T,K_{n,0}\right)\right\}|<\infty . \end{array}$$

From Equation (A18), we complete the proof. ☐

**Proof** **of** **Corollary** **2.** The proof is similar to that for Corollary 1, using the results in Theorem 2. ☐

## Appendix B. List of Notations and Symbols

- $A_{n}$: $k\times (p_{n}+1)$ matrix in hypotheses Equations (8) and (14)
- c: k dimensional vector in $H_{1n}$ in Equation (14)
- F(·): link function
- *G*: bias-correction term in “robust-BD”
- G: limit of $A_{n}A_{n}^{T}$, i.e., $A_{n}A_{n}^{T}\overset{n\to \infty }{⟶}G$
- $H_{n}$: $H_{n}=\;E_{K_{n,0}}\left\{p_{2}\left(Y;{\tilde{X}}_{n}^{T}{\tilde{\beta }}_{n,0}\right)w\left(X_{n}\right){\tilde{X}}_{n}{\tilde{X}}_{n}^{T}\right\}$
- IF(·;·,·): influence function
- *J*: an arbitrary distribution in the contamination of Equation (10)
- $K_{n,0}$: true parametric distribution of $Z_{n}$
- $K_{n,\epsilon }$: $K_{n,\epsilon }=\left(1-\frac{\epsilon }{\sqrt{n}}\right)K_{n,0}+\frac{\epsilon }{\sqrt{n}}J$, ϵ-contamination in Equation (10)
- $K_{n}$: empirical distribution of ${\left\{{Z_{n}}_{i}\right\}}_{i=1}^{n}$
- $\ell_{K}\left(\cdot \right)$: expectation of *robust-BD* in Equation (11)
- m(·): conditional mean of *Y* given $X_{n}$ in Equation (1)
- *n*: sample size
- $p_{n}$: dimension of β
- $p_{i}\left(\cdot ;\cdot \right)$: *i*th order derivative of *robust-BD*
- q(·): generating *q*-function of BD
- T(·): vector, a functional of estimator in Equation (12)
- $U_{n}$: $U_{n}=A_{n}H_{n}^{-1}\Omega_{n}H_{n}^{-1}A_{n}^{T}$
- V(·): conditional variance of *Y* given $X_{n}$ in Equation (2)
- $W_{n}$: Wald-type test statistic in Equation (9)
- w(·): weight function
- $X_{n}$: explanatory variables
- *Y*: response variable
- $Z_{n}={\left(X_{n}^{T},Y\right)}^{T}$
- α(·): level of the test
- β(·): power of the test
- ${\tilde{\beta }}_{n,0}$: true regression parameter
- $\Delta_{z_{n}}$: probability measure which puts mass 1 at the point $z_{n}$
- ϵ: amount of contamination in Equation (10), positive constant
- $\psi_{\mathrm{RBD}}\left(\cdot ;\cdot \right)$: score vector in Equation (7)
- $\Omega_{n}$: $\Omega_{n}=\;E_{K_{n,0}}\left\{p_{1}^{2}\left(Y;{\tilde{X}}_{n}^{T}{\tilde{\beta }}_{n,0}\right)w^{2}\left(X_{n}\right){\tilde{X}}_{n}{\tilde{X}}_{n}^{T}\right\}$
- $\rho_{q}\left(\cdot ,\cdot \right)$: *robust-BD* in Equation (4)
